# Double-Scale Adaptive Transmission in Time-Varying Channel for Underwater Acoustic Sensor Networks

**DOI:** 10.3390/s21062252

**Published:** 2021-03-23

**Authors:** Yi Cen, Mingliu Liu, Deshi Li, Kaitao Meng, Huihui Xu

**Affiliations:** 1 Electronic Information School, Wuhan University, Wuhan 430072, China; cenyi@whu.edu.cn (Y.C.); liumingliu@whu.edu.cn (M.L.); meng_kaitao@whu.edu.cn (K.M.); xuhuihui@whu.edu.cn (H.X.); 2 Collaborative Innovation Center of Geospatial Technology, Wuhan 430079, China

**Keywords:** adaptive transmission, double-scale channel estimation, underwater acoustic sensor networks, time-varying communication channel

## Abstract

The communication channel in underwater acoustic sensor networks (UASNs) is time-varying due to the dynamic environmental factors, such as ocean current, wind speed, and temperature profile. Generally, these phenomena occur with a certain regularity, resulting in a similar variation pattern inherited in the communication channels. Based on these observations, the energy efficiency of data transmission can be improved by controlling the modulation method, coding rate, and transmission power according to the channel dynamics. Given the limited computational capacity and energy in underwater nodes, we propose a double-scale adaptive transmission mechanism for the UASNs, where the transmission configuration will be determined by the predicted channel states adaptively. In particular, the historical channel state series will first be decomposed into large-scale and small-scale series and then be predicted by a novel *k*-nearest neighbor search algorithm with sliding window. Next, an energy-efficient transmission algorithm is designed to solve the problem of long-term modulation and coding optimization. In particular, a quantitative model is constructed to describe the relationship between data transmission and the buffer threshold used in this mechanism, which can then analyze the influence of buffer threshold under different channel states or data arrival rates theoretically. Finally, numerical simulations are conducted to verify the proposed schemes, and results show that they can achieve good performance in terms of channel prediction and energy consumption with moderate buffer length.

## 1. Introduction

In recent years, the development of underwater acoustic sensor networks (UASNs) has boosted a wide range of emerging applications, such as ocean observation, ecosystem monitoring, disaster warning, etc. [1,2,3]. Compared with terrestrial wireless sensor networks, the transmission in UASNs suffers from low data rates due to large propagation attenuation, limited bandwidth, and time-varying channels [4]. Generally, the transmission data rate highly depends on the selection of modulation method, coding rate, and transmission power, which will be referred to as transmission configuration in this paper. As battery replacement or charging is quite difficult in the underwater environment, in order to improve the transmission efficiency, the energy cost and data rate should be jointly optimized in UASNs.

**Figure 1:**
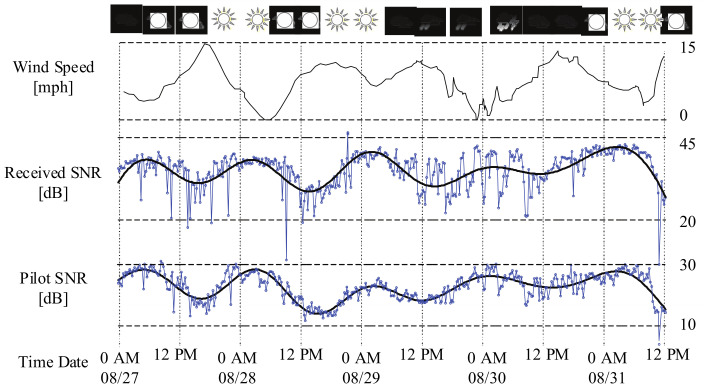
Keweenaw Waterway experiment: Average signal-to-noise rates (SNR) at the receiver.

Given specific transmission configuration, the energy efficiency of data transmission will be affected by different channel states. Considering the time-varying communication channels in UASNs, it is crucial to learn the channel variation characteristics for determining the optimized transmission configuration. The underwater channels could be affected by various environmental factors, including water temperature, wind speed, tidal, ocean swell, and so on. These natural phenomena occur at different time scales, such as seasonal, diurnal, minutes, and seconds [5,6,7,8]. The overlay of these time scales forms the complex fluctuation in underwater communication channels. Figure 1 presents an example of the channel states in an underwater communication experiment [9]. Obviously, the fluctuation of the received signal-to-noise rate (SNR) is roughly consistent with that of the wind speed.

Based on the above observation, we propose to take advantage of the historical channel state series and analyze the fluctuation characteristics for channel state prediction, so that an optimized configuration scheme can be derived and the energy efficiency of data transmission would be enhanced accordingly. However, it is quite challenging to schedule the optimal configuration scheme dynamically due to the following reasons. First of all, the prediction of underwater channels is difficult as they are impacted by multiple factors, such as underwater geology, salinity and depth, environmental factors (ocean current, surface wind, sea wave, solar radiation, etc.), human-being activities, and fish behaviors, and it is hard to model the fluctuation by just using long time-scale historical channel state data or just using short time-scale cycle information. Secondly, to improve the overall performance of underwater sensor networks, the transmission rate and energy cost should be jointly optimized based on the predicted channel states. Thirdly, considering the limited computational and storage capability of underwater sensor nodes, how to balance the network quality and reducing complexity is essential. For example, even though the quality of network service, such as network throughput, can be improved with a fine-grained transmission scheme, the frequent change of transmission modes might cause extra computational complexity and energy cost.

In the literature, adaptive transmission according to channel state has become a hot topic. The rule-based adaptive modulation and coding (AMC) methods usually utilize fixed thresholds for transmission mode determination [1,10,11,12]. These methods are easy for implementation and could be used in the resource-constrained networks, while the threshold selection should be carefully designed. Learning strategies [13,14,15,16] have been proposed to provide intelligent transmission decisions to characterize the dynamic communication channel, and the Markov chain is widely adopted. However, the state space would be enlarged with a large channel fluctuation amplitude, which may increase the training burden and computational complexity. Optimization [17,18] schemes have also been investigated for adaptive transmission, but fine-grained optimization will lead to greater computational complexity.

To provide energy-efficient transmission for UASNs, we propose a double-scale adaptive transmission mechanism in this paper. The historical channel state series will first be decomposed into large-scale and small-scale series; then, real-time channel state can be predicted according to different fluctuation features with each time scale. In order to solve the problem of long-term transmission configuration with modulation and coding mode selection, an energy-efficient transmission algorithm is designed on the basis of channel state prediction. The contributions of this paper can be summarized as follows:

- To improve the energy efficiency and reliability of data transmission, we propose a double-scale adaptive transmission mechanism for UASNs. Specifically, the historical channel state series is used for channel state prediction, and then the transmission mode is determined adaptively.
- To balance the accuracy and computational complexity of channel states prediction, we propose to decompose the channel state series with two different time scales. For the large-scale channel state, a *k*-nearest neighbor algorithm with sliding window is designed to predict the fluctuation tendency, and then a small-scale channel state prediction algorithm is developed to enhance the accuracy.
- To determine the specific configuration of data communication in UASNs, we design an energy-efficient transmission algorithm. In particular, the long-term modulation and coding problem is formulated and optimized with the constraint of limited energy cost.

The paper is organized as follows. In Section 2, the related works are presented. Section 3 illustrated the framework of our proposed transmission method, and then the double-scale channel prediction and transmission scheduling are given in Section 4 and Section 5, respectively. Quantitative performance analysis and computational complexity are presented in Section 6. Simulation results are analyzed in Section 7, and, finally, the paper is concluded in Section 8.

## 2. Related Works

In this section, existing prediction methods and adaptive transmission methods designed for the dynamic communication environment are briefly reviewed.

### 2.1. Channel State Prediction

To resolve the inefficient and unstable communication caused by time-varying channel conditions, prediction methods for future channel states have been widely studied in recent years. Specifically, existing works can be classified into direct prediction and decomposition-based prediction methods, and direct prediction methods include linear and non-linear channel prediction methods.

Linear prediction models have been used for channel prediction in wireless communication. The auto-regressive (AR) model was proposed in Reference [19] to predict the channel impulse response, which was expressed as a linear combination of current and past channel states. Liu et al. [20] employed a channel prediction framework based on auto-regressive predictors to exploit both the spatial and temporal correlations among antennas. An improved adaptive Kalman estimator was proposed in Reference [21] for the adaptive fading channel. A recursive approximated structure with filter bank and discrete cosine transform was proposed in Reference [22] for channel prediction. For underwater channel state forecast, linear prediction models, such as statistical analysis [23] and exponential moving average (EMA) [24], were utilized, as well. In Reference [11], auto-regression was used every several symbols for the adaptive modulation of underwater communication. Zhang et al. [25] proposed an adaptive channel prediction scheme based on the exponential weighted recursive least square (EWRLS) algorithm, which used current and past estimated channel parameters in the delay-Doppler domain.

Compared with linear prediction methods, non-linear channel prediction can achieve a smaller mean square error (MSE). A support vector machine (SVM) was employed in Reference [26] to predict channel state in airplane cabin scenarios. In Reference [27], an echo state network (ESN) was utilized for fast channel prediction in Ricean fading scenarios, which obtained smaller prediction error than previous designs. Tripathi et al. [28] proposed novel channel prediction frameworks by using stochastic modeling, as well as data-driven learning of channel variability. A deep learning-based algorithm was proposed in Reference [29] to predict future channel state information (CSI) and received signal levels. Due to the complex fluctuation of underwater channel state with environmental noise, non-linear prediction methods can significantly improve accuracy [30]. Diao et al. [30] introduced a channel prediction model based on nearest neighbor regression for underwater acoustic networks, where a fast search algorithm and a statistical storage compression method were used to optimize the time and space complexity of the prediction scheme. However, non-linear prediction methods usually show high computational complexity, while the computational capacity of underwater sensor nodes is limited.

Due to the advantage of significant prediction accuracy, decomposition-based prediction methods have become promising solutions for channel prediction in the most recent years. Long et al. [31] introduced multi-resolution wavelet analysis to predict the received signal strength in the fast varying wireless environment. In Reference [5], the change process of underwater acoustic channel state was modeled as the sum of an environmental process affected by measurable environmental parameters and a Markov process explaining the contribution of unknown physical mechanisms. Based on the historical channel state sequence and recorded environmental parameters, a recursive algorithm was proposed to estimate the combination coefficient of the environmental parameters and the Markov process for channel prediction. The decomposition-based prediction methods show good performance [32] and the reason is as follows. From the perspective of divide and conquer, the prediction based on decomposition can enhance the prediction ability of the model [33], as the original non-linear and non-stationary sequence is decomposed into a finite number of subsequences, which have simpler frequency components. Thus, the difficult prediction task is divided into several relatively easy subtasks [34].

### 2.2. Adaptive Data Transmission

Adaptive transmission schemes have been studied in terrestrial wireless communications to improve communication performance. Huang [35] investigated cross-layer scheduling and power control combined with adaptive modulation for wireless ad hoc networks. Reinforcement learning (RL) has also been used for adaptive transmission in terrestrial wireless communication. A transmission scheduling strategy based on deep Q-learning (DQN) was proposed in Reference [36] to maximize the system utility composed of throughput, buffer pressure, and power consumption for the cognitive Internet of Things. Li et al. [37] studied a throughput maximization problem based on deep Q-learning in a wireless communication system with energy harvesting and energy limited transmitter. For hybrid satellite-terrestrial relay networks, the performance of adaptive transmission was investigated in Reference [38] with a decode-and-forward relay. Ekerete et al. [39] investigated adaptive margins for AMC in broadband satellite links during the actual rain event. To increase system throughput and improve transmission efficiency, an adaptive coding transmission (ACT) scheme was proposed in Reference [40] over the satellite-terrestrial channel based on the analog fountain code (AFC), achieving a seamless performance across all channel states.

Underwater acoustic communication surfs from the following difficulties: large amplitude of channel variation, large propagation delay, narrow bandwidth, and low bit rate. Compared with terrestrial wireless sensor networks, the design of UASNs is more challenging.

Adaptive transmission schemes based on rules have been investigated for underwater acoustic communication. Wan et al. [1] proposed an AMC system to maximize the transmission rate with a given transmission power for underwater communication using orthogonal frequency-division multiplexing (OFDM). And the system utilized a finite number of transmission modes which were switched based on the effective signal-to-noise ratio (ESNR). Different modulation methods were used in Reference [10] for opportunistic cooperative transmission of underwater networks under various environmental conditions to achieve the best compromise between robustness and data rate. Kuai et al. [11] proposed a fixed threshold adaption algorithm and the required SNR of different modulation methods were presented to meet the target bit error rate (BER). An adaptive OFDM transmission system was proposed in Reference [12] based on the SNR while maintaining a certain bit error rate (BER). Although these rule-based schemes are convenient to operate, fixed thresholds are not applicable for complex channel fluctuations and varying data transmission requirements, which will result in performance degradation.

Some researchers investigated adaptive transmission based on learning algorithms for underwater acoustic communication. In Reference [41], Xiao et al. proposed an RL-based antijamming relay scheme, as well as a deep-RL-based relay scheme, for UWSNs to optimize the relay mobility and power allocation. Tomasi et al. [13] designed two transmission schemes based on dynamic programming (DP) and a heuristic strategy to send a specified number of packets before a deadline while minimizing transmission attempts. To maximize the expected total amount of delivered data in finite time, energy management was investigated in Reference [14] for underwater acoustic nodes. And a stochastic dynamic programming algorithm was used to solve the optimal solution in energy allocation and a suboptimal algorithm was presented with reduced complexity. Reinforcement learning can be used to derive optimal actions for adaptive transmission in dynamic environments. A RL-based protocol was developed in Reference [42] for underwater acoustic communication to minimize a cost function which combined delay and energy consumption and ensure reliable transmission. Wang et al. [15] formulated the adaptive transmission problem as a partially observable Markov decision process for underwater acoustic communication systems. And a model-based reinforcement learning strategy was used to develop an online algorithm, which could derive the optimal transmission actions to minimize a long-term cost. A reinforcement learning-based adaptive modulation and coding algorithm was proposed in Reference [16] for underwater communications based on the network states. However, the learning-based methods have high training complexity and usually need a long time to converge. Moreover, the reward function should be designed reasonably, which is also a problem to be studied.

Some optimization-based adaptive transmissions have been studied for underwater acoustic communication. In Reference [17], adaptive OFDM modulation and power allocation were investigated to maximize the throughput while maintaining a target average bit error rate (BER). Two schemes were developed with different levels of adaptivity based on a greedy algorithm: One scheme could adjust only the modulation levels and adopted a unified power allocation, while the other scheme could adjust both the modulation levels and the power allocated to each subcarrier. Wang et al. [18] investigated the energy efficiency maximization problem in underwater acoustic channels with periodic dynamics. Assuming that the future channel states were known, a water-filling algorithm was designed to schedule the optimal transmission actions. For channels with causal knowledge, the optimal actions were scheduled based on the predicted channel states and the packet queue condition. However, the computational complexity was relatively high to schedule actions for all time slots.

## 3. Double-Scale Adaptive Transmission Mechanism for UASNs

### 3.1. System Model

**Figure 2:**
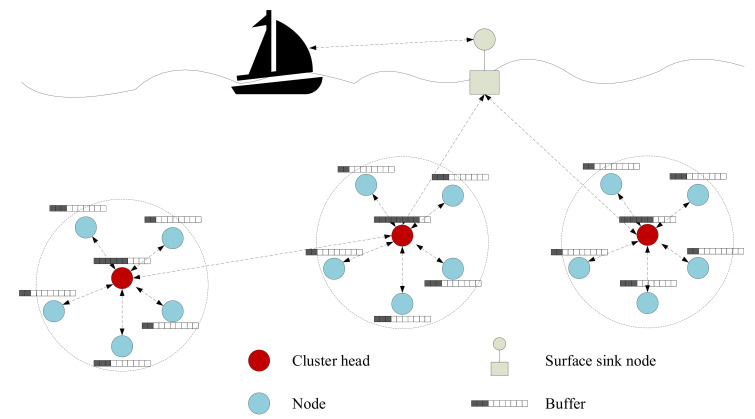
Multi-hop clustered underwater acoustic sensor network.

Most UASNs can be modeled as a multi-hop clustered network as shown in Figure 2. The clustered UASN consists of a surface sink node and underwater sensor nodes. Generally, the sensor nodes can be classified into cluster headers (CH) or cluster members (CM). The CM nodes gather environmental information and forward the data to its CH. Then, each CH transmits collected data to the sink node through single-hop or multi-hop transmission. The transmission slots of all sensors are scheduled based on media access control (MAC) algorithms, such as Time Division Multiple Access (TDMA). Each node sends at the scheduled slot, and the time interval between two transmissions is assumed to be equal.

The signal-to-noise ratio (SNR) can be considered as an indicator of the channel state, which is defined as the received SNR corresponding to a transmission power of one unit. We assume that the channel state remains stable in each slot and would change in the next slot. In each time slot, some packets arrive at the transmitter node and enter the data buffer. $B_{max}$ denotes the buffer capacity of the transmitter. At the beginning of slot *i*, the existing buffer length is $B_{i}$. Let the number of arriving packets be $g_{i}$, and the number of transmitted packets be $l_{i}$, the new buffer length can be expressed as

(1) $$B_{i+1}=min\left\{max\left\{B_{i}-l_{i},0\right\}+g_{i},B_{max}\right\}.$$

The amount of arrival data in the future can be predicted by recursive least squares (RLS) filter [43] and artificial neural network [44]. So, in this paper, for the sake of simplicity, it is assumed that the sensors collect sensory data at certain time intervals for a period of time so that each node can know the data arrival rate in the future period.

The set of modulation and coding modes is defined as $\left\{m_{0},m_{1},\cdots ,m_{M}\right\}$, which is sorted in ascending order of transmission rate. In this set, $m_{0}$ means stop transmission and $m_{M}$ yields max transmission rate. $P_{max}$ denotes the maximum transmission power of the transmitter and *M* represents the number of modulation and coding modes.

### 3.2. Underwater Acoustic Channel Model

Throughout this article, a shallow-water acoustic propagation environment is considered. According to the principle of underwater acoustic, the signal-to-noise ratio of an underwater acoustic signal at the receiver can be calculated using the passive sonar equation as follows [45]:

(2) $$\begin{array}{c} SNR\;=\;SL\;-PL\;-\;NL+DI\geq DT,\;(in\;\mathrm{dB}\;\mathrm{re}\;\mu \mathrm{Pa}), \end{array}$$

where SL is the source level, PL is the transmission loss, NL is the noise level, DI is the directive index, and DT is the detection threshold of the sonar for specific modulation and coding mode. All these quantities are in dB re μPa, where the reference value of 1 μPa equals to $0.67\times 10^{-18}$ Watts/m${}^{2}$[45]. For the convenience of expression, the notation dB is used to signify dB re μPa in the rest of this paper. The path loss for a signal over distance *d* is given by Reference [46] as

(3) $$\begin{array}{c} PL=f_{s}\cdot 10\mathrm{lg}d+\alpha \cdot d\times 10^{-3} \end{array},$$

where $f_{s}$ is the spreading factor, which can be taken as 1.5 for a practical scenario, and α is the absorption coefficient, which can be modeled by Thorp’s formula [45] as follows:

(4) $$\begin{array}{c} \alpha =\frac{0.11f^{2}}{1+f^{2}}+\frac{44f^{2}}{4100+f^{2}}+\frac{2.75f^{2}}{10^{4}}+0.003 \end{array},$$

where α is given in dB/km, and *f* is in kHz. The power spectrum density of the ambient noise in the ocean can be calculated by

(5) $$\begin{array}{c} NL=N_{turb}\left(f\right)+N_{ship}\left(f\right)+N_{wave}\left(f\right)+N_{ther}\left(f\right) \end{array},$$

where $N_{turb}\left(f\right)$, $N_{ship}\left(f\right)$, $N_{wave}\left(f\right)$, and $N_{ther}\left(f\right)$ in dB per Hz represent the turbulence noise, the shipping noise, the waves noise, and the thermal noise, respectively. These noises can be modeled by the following empirical formulas [46]:

(6) $$\begin{array}{c} 10log\left(N_{turb}\left(f\right)\right)=17-30logf, \end{array}$$

(7) $$\begin{array}{c} 10log\left(N_{ship}\left(f\right)\right)=40+20\left(s-0.5\right)+26logf-60log\left(f+0.03\right), \end{array}$$

(8) $$\begin{array}{c} 10log\left(N_{wave}\left(f\right)\right)=50+7.5\sqrt{w}+20logf-40log\left(f+0.4\right), \end{array}$$

(9) $$\begin{array}{c} 10log\left(N_{ther}\left(f\right)\right)=-15+20logf, \end{array}$$

where *s* and *w* denote the shipping activity factor and wind speed, respectively. The channel state $h_{t}$ is defined as the received signal-to-noise ratio corresponding to a source level $SL_{0}$ with a transmission power of one unit. When the source level is $SL_{1}$ and the signal-to-noise ratio at the receiver is $SNR_{1}$, channel state is recorded as

(10) $$\begin{array}{c} h_{t}=SNR_{1}-SL_{1}+SL_{0} \end{array}.$$

According to the above formula, channel state can vary with the dynamic environmental factors. The underwater channels could be affected by various environmental factors, such as water temperature, wind speed, tidal, ocean swell, and so on, and these natural phenomena occur at different time scales, such as seasonal, diurnal, minutes, and seconds. These environmental factors result in complex fluctuation of the underwater acoustic channel on various time scales. Specially, the well-known “afternoon effect” caused by the diurnal and seasonal change in water surface temperature can result in the diurnal and seasonal change in the signal and noise strength [9]. As a result, the fluctuation characteristics of channel state can be analyzed for channel state prediction, and an optimized configuration scheme can be derived to improve the energy efficiency of data transmission.

### 3.3. Adaptive Transmission Framework

**Figure 3:**
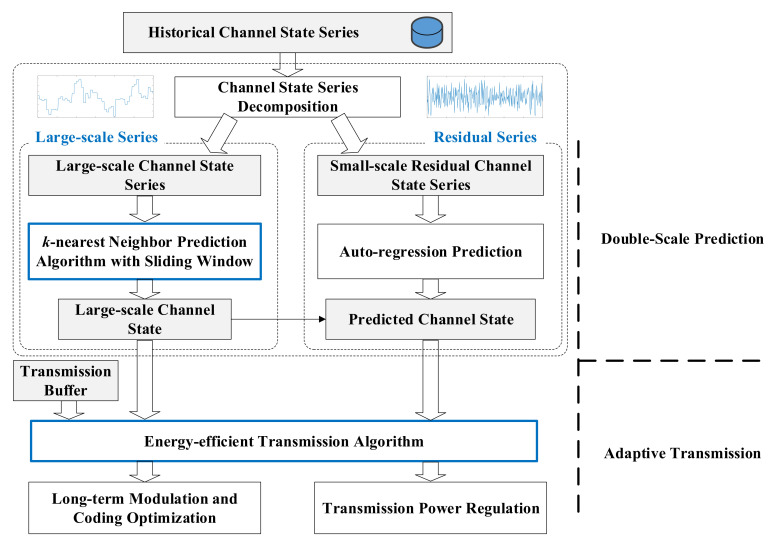
Double-scale adaptive transmission mechanism.

To improve energy efficiency and reliability of underwater acoustic communication, a double-scale adaptive transmission mechanism is proposed based on the fluctuation characteristics of underwater channel, as shown in Figure 3. The transmission framework consists of two parts, namely double-scale prediction and adaptive transmission. In double-scale prediction, the historical channel state series is decomposed into large-scale and residual series, which will be predicted by a novel *k*-nearest neighbor search algorithm with sliding window and auto-regressive algorithm, respectively. In adaptive transmission, an energy-efficient transmission algorithm is designed to solve the problem of long-term modulation and coding optimization.

**Figure 4:**
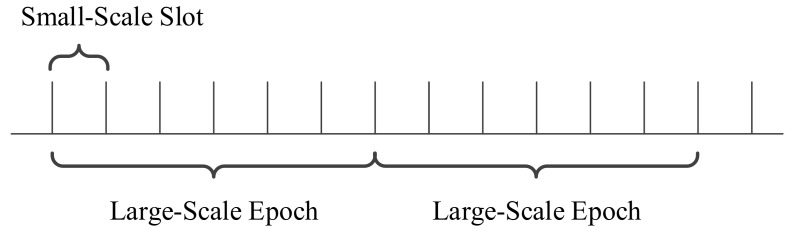
Large-scale epoch and small-scale slot.

To reduce the complexity of scheduling, the transmitter of a CH or a CM node uses the same modulation and coding mode in a large-scale time. In this way, the modulation and coding mode only needs to be scheduled once for each large-scale period. For the convenience of description, epoch and slot are used to refer to large-scale and small-scale time, respectively. A large-scale epoch consists of $N_{large}$ slots, as shown in Figure 4.

In detail, the large-scale decision aims to predict large-scale channel states and determine the modulation and coding mode for each large-scale epoch. As it is difficult to predict the future channel state in each slot, the average channel state of each large-scale epoch in the future is predicted for schedule. Large-scale channel state is defined as the average channel state in a large-scale epoch. Then, based on the present buffer state and predicted large-scale channel states, the modulation and coding mode in each future large-scale epoch will be scheduled.

Then, given the scheduled modulation and coding mode, the small-scale decision operates to predict the channel state of the next slot and control the transmission power. To improve the accuracy of prediction, the channel state in each slot is predicted based on series decomposition.

In a large-scale epoch, the average channel change should be less than a certain threshold $D_{t}$, so that the same modulation and coding mode can be allocated for the slots in this large-scale epoch. Thus, the algorithm calculates the average time $T_{large}$ required for channel state change $D_{t}$ and takes $T_{large}$ as the length of large-scale epoch. $D_{t}$ is given as

(11) $$D_{t}=\frac{h_{max}-h_{min}}{2M},$$

where $h_{max}$ and $h_{min}$ are the best channel state and worst channel state, respectively, and *M* is the number of modulation and coding modes.

At the beginning of a slot, packets are sent by the transmitter. Before the end of the slot, an acknowledgment packet is sent from the receiver to the transmitter via an error-free channel, including the number of packets successfully decoded and the received signal-to-noise ratio (SNR) of each packet.

## 4. Double-Scale Channel State Prediction

In this section, the proposed prediction algorithms for the large-scale and small-scale channel states are presented, respectively.

### 4.1. Large-Scale Channel State Prediction

To predict large-scale channel states, a *k*-nearest neighbor algorithm with sliding window is designed based on subsequence matching. Furthermore, in order to reduce the computational complexity, the length of time series to be reserved is calculated according to the characteristics of channel fluctuation.

#### 4.1.1. *k*-Nearest Neighbor Prediction Algorithm with Sliding Window

**Figure 5:**
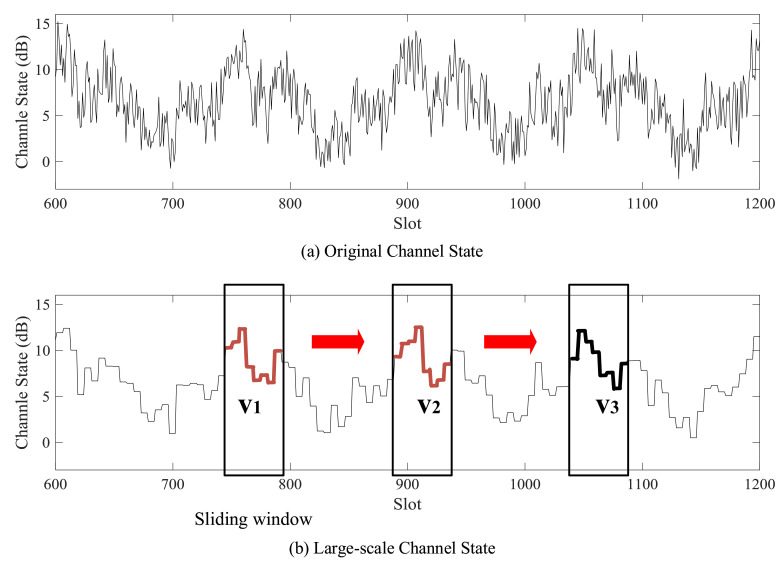
Channel state and large-scale channel state prediction method. (**a**) Original channel state. (**b**) Large-scale channel state and diagram of *k*-nearest neighbor algorithm with sliding window. $v_{3}$ is the test vector. $v_{1}$ and $v_{2}$ are nearest neighbors chosen from training vectors.

Large-scale channel state is represented as the average channel state in a large-scale epoch, as shown in Figure 5. Large-scale channel state of epoch *j* is

(12) $$\begin{array}{c} h_{j}^{L}=\frac{1}{N_{large}}\sum_{i=1}^{N_{large}}h_{i}^{S}, \end{array}$$

where $N_{large}$ is the number of slots in a large-scale epoch. $h_{i}^{S}$ is the channel state of the *i*-th slot during the epoch *j* in dB. For the convenience of expression, the notation $h_{j}$ is used to signify $h_{j}^{L}$ in Section 4.1.

Assuming that the current time is in the *u*-th large-scale epoch, and the stored historical series of large-scale channel state can be represented as

(13) $$H^{L}=\left[h_{1},h_{2},\cdots ,h_{u}\right],$$

where $h_{i}$ denotes the large-scale channel state in epoch *i*.

The scheme of large-scale channel prediction is shown in Algorithm 1. The input elements of the algorithm include the training set which consists of training vectors and a test vector. And the output of the algorithm is the predicted large-scale channel state after *v*-th epochs.

The training set $Y=[S_{n},S_{n+1},\cdots ,S_{u-v}]$ contains a group of training vectors and their labels. Training vectors are obtained from $H^{L}$ by a sliding window with a length of *n*, which is the order of the prediction model, as shown in Figure 5b. The label is the *v*-th value after the corresponding vector in $H^{L}$. Training vector $S_{i}$ and its label $h_{i+v}$ is given as

(14) $$S_{i}=\left[h_{i-n+1},h_{i-n+2},\cdots ,h_{i-1},h_{i}\right]∼h_{i+v},i\in \left[n,u-v\right].$$

Test vector $S_{u}$ contains channel state of *n* large-scale epochs before current large-scale epoch,

(15) $$S_{u}=\left[h_{u-n+1},h_{u-n+2},\cdots ,h_{u-1},h_{u}\right].$$

Firstly, each training vector is matched according to its last value. In detail, training vector $S_{i}$ is selected if the last values of $S_{i}$ and $S_{u}$ are similar, meeting the following condition,

(16) $$|h_{i}-h_{u}|\leq \frac{h_{max}-h_{min}}{M}.$$

**Algorithm 1:** large-scale channel state prediction**Input**: training set Y, test vector $S_{u}$ **Output**: predicted large-scale channel state ${\hat{h}}_{u+v}$ 1**for***vector*$S_{i}$*in training set*Y**do**2
     **if** *last value of* $S_{i}$ *and* $S_{u}$ *are similar* **then**
3
        Calculate distance of $S_{i}$ and $S_{u}$4
     **end if**
5**end for**6Choose nearest *k* vectors with labels7
Calculate weight of chosen vectors8
Obtain predicted channel state 

If this condition is not satisfied, $S_{i}$ is filtered out. Most of the training vectors are filtered out by this condition, so the computational complexity is greatly reduced.

To reduce the computational complexity, the L1 metric is calculated as the distance between $S_{i}$ and $S_{u}$. The L1 metric is represented as

(17) $$D_{S_{i}S_{u}}=\left|\right|S_{i}-S_{u}{\left|\right|}_{1}.$$

Then, *k* nearest neighbors $\left[c_{1},c_{2}\ldots c_{k}\right]$ are chosen with labels $\left\{h_{v}^{c_{1}},h_{v}^{c_{2}},\cdots ,h_{v}^{c_{k}}\right\}$ as *k* prediction values. For example, in Figure 5, nearest neighbors $v_{1}$ and $v_{2}$ are chosen for test vector $v_{3}$.

Inverse distance weight of chosen vector $c_{j}$ is calculated as

(18) $$w_{c_{j}}=\frac{1/D_{c_{j}S_{u}}}{\sum_{m=1}^{k}1/D_{c_{m}S_{u}}}.$$

Finally, the predicted channel state is calculated with inverse distance weight, which is represented as

(19) $${\hat{h}}_{u+v}=\sum_{m=1}^{k}w_{c_{m}}h_{v}^{c_{m}}.$$

#### 4.1.2. Calculation of Stored Series Length

Since historical channel state series increases with time and will consume large storage space and increase the prediction complexity, we propose to take advantage of the fluctuation features. Considering the certain changing cycle in a long-time period, only a few cycles of historical channel state series need to be stored to reduce the amount of storage and accelerate the prediction speed.

To calculate the spectrum of historical channel state series, the Fourier transform is used. Then, the frequency $f_{max}$ with maximum amplitude is selected. The time $T_{p}$ corresponding to $f_{max}$ represents the characteristic time of channel fluctuation. The historical channel state series with a length of $T_{p}$ can reflect the characteristics of channel fluctuation. Thus, the historical channel state series with a length of $\beta T_{p}$ is stored for channel prediction, β>1.

### 4.2. Small-Scale Channel State Prediction

To accurately regulate transmission power in time-varying acoustic channels, a decomposition-based prediction algorithm is proposed.

#### 4.2.1. Small-Scale Channel Fluctuating Features

Due to the complex fluctuation of underwater acoustic channel state, direct prediction usually suffer from large prediction error. Over-estimation of the channel state will lead to packet loss, while under-estimation will lead to energy efficiency deterioration.

To improve the accuracy of prediction, a decomposition-based prediction model is proposed. The advantage of this model is that it adopts a series decomposition method with low complexity to improve the prediction accuracy, and a large-scale channel state only needs to be predicted once in a large-scale epoch.

**Figure 6:**
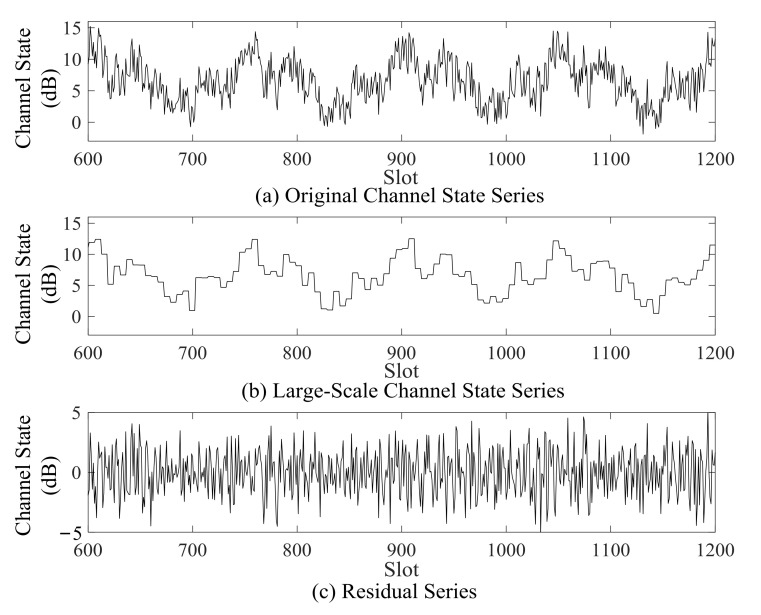
Channel state series decomposition.

Firstly, the large-scale channel state $H^{L}$ is subtracted from the original channel state series H to obtain the high frequency residual series $H^{re}$, as shown in Figure 6.

(20) $$\begin{array}{c} H^{re}=H-H^{L} \end{array}.$$

The residual series can represent small-scale fluctuating features of the underwater acoustic channel. Although prediction models based on other decomposition methods, such as discrete wavelet decomposition [31] and empirical mode decomposition [32,33,34], can further improve the prediction accuracy, the advantage of the decomposition method in this paper is its lower complexity.

#### 4.2.2. Residual Series Prediction

Then, after decomposition of the original channel state series, the auto-regression (AR) model is used to predict the residual series due to the low computational complexity. The AR prediction model is presented as

(21) $$\begin{array}{c} {\hat{h}}_{t+1}^{re}=\sum_{i=1}^{L_{AR}}a_{i}h_{t-L_{AR}+i}^{re}+b_{t+1}. \end{array}$$

${\hat{h}}_{t+1}^{re}$represents the predicted value of residual series in slot t+1, and $h_{t}^{re}$is the value of the residual series in slot *t*. $L_{AR}$ denotes the prediction order. $a_{i}$ represents the *i*-th coefficient of AR prediction model and and $b_{t+1}$ is a noise term. The coefficients of the AR model can be calculated by the least squares algorithm [5,11,25].

Finally, given the predicted large-scale channel state values ${\hat{h}}_{t+1}^{L}$ in Section 4.1.2, and the prediction value of residual series ${\hat{h}}_{t+1}^{re}$, the predicted channel state of the next slot is

(22) $$\begin{array}{c} {\hat{h}}_{t+1}={\hat{h}}_{t+1}^{L}+{\hat{h}}_{t+1}^{re}. \end{array}$$

## 5. Energy-Efficient Transmission Algorithm

Based on the predicted large-scale channel states, an energy-efficient transmission algorithm is proposed to schedule the modulation and coding modes.

### 5.1. Problem Formulation

Since the large-scale channel states in the future have been predicted, transmission configuration can be scheduled. The objective of the energy efficiency optimization problem is to minimize the ratio of energy cost to the amount of data successfully delivered, which can be formulated as:

(23) $$min\frac{\sum_{k=1}^{N_{sch}}E_{k}^{m}\left({\hat{h}}_{k}\right)}{\sum_{k=1}^{N_{sch}}R_{k}^{m}\left({\hat{h}}_{k}\right)},$$

subject to

(24) $$\sum_{k=1}^{N_{sch}}R_{k}^{m}\left({\hat{h}}_{k}\right)\geq A_{s},$$

(25) $$0<P_{k}\leq P_{max},$$

(26) $$m\in \{m_{0},m_{1},\cdots ,m_{M}\}.$$

${\hat{h}}_{k}$ represents the predicted channel state of large-scale epoch *k*. $R_{k}^{m}\left({\hat{h}}_{k}\right)$ is the expected amount of successfully transmitted data with *m* modulation and coding mode at the large-scale epoch *k*. $E_{k}^{m}\left({\hat{h}}_{k}\right)$ denotes the expected energy cost of *m* transmission mode at the large-scale epoch *k*. $A_{s}$ denotes the amount of data that needs to be sent in the planned time. $P_{k}$ represents the transmission power in large-scale epoch *k*. $N_{sch}$ is the number of scheduled large-scale epochs.

Constraint (24) means that the expected amount of successfully delivered data should be larger than the amount of data requested to be transmitted in the scheduling period. Constraint (25) means that the transmission power should not exceed the maximum power of the transmitter. Constraint (26) means that the transmission mode should be selected from the available modulation and coding modes.

In order to calculate the amount of data to be sent, a threshold $B_{c}$ is set to distinguish the buffer states. When the data queue exceeds this threshold, the transmitter will transmit as many packets as possible to make the data queue length lower than this threshold. $M_{max}$ is the max transmitted bits of all modulation and coding modes in a large-scale epoch. STP is the successful transmission probability under the current channel prediction accuracy, which is obtained by statistical method. Thus, $M_{max}*STP$ represents the maximum transmission capacity. According to the data arrival rate, transmission requirements can be divided into three cases:

(1) When the bits arrival rate is less than the maximum transmission capacity, and the buffer size is less than the buffer threshold, the amount of successfully transmitted bits should be more than the expected arrival bits.
(2) When the bits arrival rate is less than the maximum transmission capacity, and the buffer size is greater than the buffer threshold, the amount of successfully transmitted bits should be more than the expected arrival bits plus a certain proportion ε of the buffer length, 0<ε<1.
(3) When the bits arrival rate is greater than the maximum transmission capacity, the message should be sent according to the maximum transmission capacity.

As mentioned above, the amount of data required to be transmitted is given as

(27) $$A_{s}=\left\{\begin{array}{cccc} \; & \lambda *L, & \; & \lambda \leq M_{max}*STP,B_{i}\leq \begin{array}{c} B_{c} \end{array},\\ \; & \lambda *L+\varepsilon *B_{i}, & \; & \lambda \leq M_{max}*STP,B_{i}>B_{c},\\ \; & M_{max}*STP*L, & \; & \lambda >M_{max}*STP. \end{array}\right.$$

λ represents the amount of arrival data in each large-scale epoch. $B_{i}$ denotes the buffer state at present.

The buffer threshold $B_{c}$ can influence the corresponding energy cost, as well as average transmission delay. The transmission delay is reduced with the decrease of buffer threshold at the cost of increasing energy consumption, while, with the increase of buffer threshold, more packets can be allowed to stay in the buffer until the channel state becomes good, which will reduce the energy consumption and increase the transmission delay. In practice, the buffer threshold can be adjusted according to the application scenarios. When the transmission is delay tolerant, and the energy consumption is more important, the buffer threshold can be set as a large value. In applications that require relatively low latency, the buffer threshold should be reduced. The impact of buffer threshold on transmission performance will be presented in quantitative analysis and simulation, which can be used to select buffer threshold.

### 5.2. Modulation Coding Method Selection

Problem (23) is an integer programming problem. This kind of problem can be solved by heuristic algorithms, such as particle swarm optimization and genetic algorithm. In this paper, an improved genetic algorithm is designed to solve the problem as shown in Algorithm 2. To accelerate the speed of obtaining the optimal solution, a rearrangement process is used.

Firstly, *G* chromosomes are generated as the initial population. Modulation and coding modes are encoded as genes, and each chromosome is in the following form,

(28) $$MC=[mc_{1},mc_{2},\ldots ,mc_{L}],$$

where $mc_{i}$ is a modulation and coding mode for epoch *i*. In order to ensure the diversity of the population, the combinations of various transmission modes are used as the initial population, while the required amount of delivered data are met.

**Figure 7:**
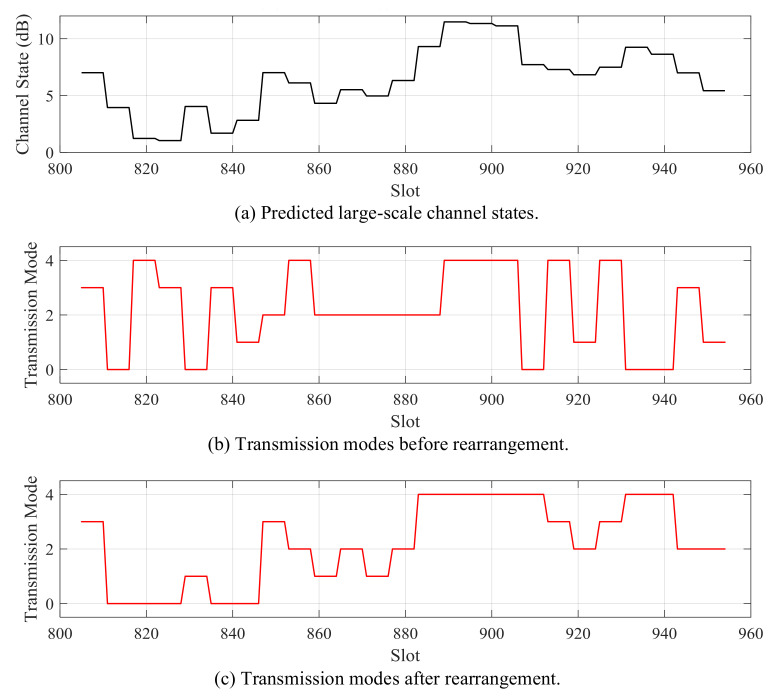
Rearrangement process for a transmission modes chromosome.

A rearrangement process is utilized on each chromosome to adjust the positions of genes according to the predicted large-scale channel states. The lower modulation coding methods are allocated to worse channel states, and higher modulation coding methods are adopted for better channel states, as shown in Figure 7. The rearrangement process accelerates the convergence speed of the algorithm.

Then, the estimated energy cost and the expected amount of delivered data will be calculated. The packet error rate (PER) can be determined based on the received SNR by using an information-theoretic approach [47] or an empirical formula estimated by real data [1]. So, according to the scheduled modulation coding methods, the predicted large-scale channel states, and target PER, the transmission power can be set. Thus, energy cost and amount of delivered data can be estimated.

For each chromosome, fitness is defined as

(29) $$fitness=\left\{\begin{array}{ccccc} \; & \frac{{\hat{R}}_{e}}{{\hat{E}}_{e}}, & \; & {\hat{R}}_{e}\geq A_{s}, & \\ \; & 0, & \; & {\hat{R}}_{e}<A_{s}. \end{array}\right.$$

${\hat{E}}_{e}$ and ${\hat{R}}_{e}$ are the estimated energy cost and the expected amount of delivered data for the scheduling time, respectively. Large fitness means that the chromosome has high energy efficiency, and the required amount of transmission data is satisfied.

*K* chromosomes with large fitness scores are preserved. (G−K)/2 pairs of chromosomes are selected with a probability according to fitness. Each pair of the selected chromosomes are crossed and mutated to produce two new chromosomes as offspring. For a pair of chromosomes, the standard crossover operation recombines them by interchanging portions of them, producing divergent solutions to explore the search space. The mutation operation is performed on a chromosome by changing an element at a random position of the chromosome. After crossover and mutation, the rearrangement process is utilized on each new chromosome.

*K* preserved chromosomes and G−K new chromosomes form the next generation of the population. As the algorithm continues and the new population evolves, the fitness scores of chromosomes improve. Finally, after several rounds of selection, crossover, and mutation, a good solution is obtained.

Before each transmission, given the scheduled modulation and coding mode for this large-scale epoch, the predicted channel state of the next slot, and target PER, the transmission power can be determined.
**Algorithm 2:** Modulation and coding mode scheduling**Input**: predicted large-scale channel state, buffer state, data arrival rate **Output**: modulation and coding modes
1
Generate initial population2
Rearrange chromosomes3**for***crosstime = 1:MaxCrossTime***do**4
     Calculate fitness
5
     Choose chromosomes with good fitness6
     Crossover and mutation7
     Rearrange new chromosomes 8**end for**9
Choose the chromosome with the best fitness 

## 6. Performance Analysis and Computational Complexity

### 6.1. Performance Analysis

In this subsection, quantitative analysis is presented about the impact of buffer threshold on communication performance, and a reasonable buffer threshold is derived for a special channel state series.

#### 6.1.1. Special Channel State Series

**Figure 8:**
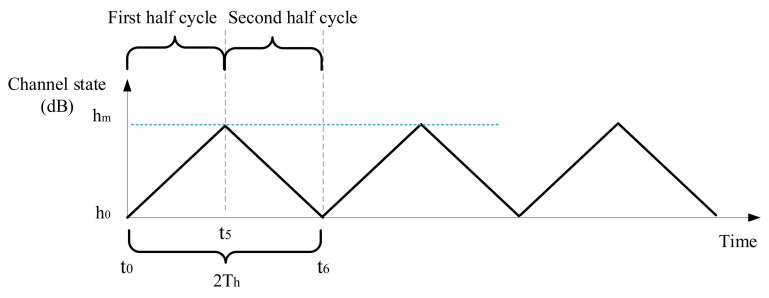
A linearly varying channel state series.

In this section, a linearly varying channel state series is presented in Figure 8, and the corresponding transmission performance will be analyzed. It is assumed that only the buffer threshold limits the data queue length, so the buffer length can grow without other restrictions. Moreover, the transmitter does not discard any data packets. Based on this situation, the transmission action of energy cost minimization will be derived. And the impacts of buffer threshold and data arrival rate on communication performance will be analyzed, in terms of energy consumption, average buffer length, and transmission delay. In Section 5.1, the buffer threshold is used to set the amount of data that needs to be sent once every $N_{sch}$ epochs, while, in this section, the buffer threshold always limits the buffer length.

Because the channel fluctuation in the first half cycle (from $t_{0}$ to $t_{5}$) is similar to that of the second half cycle (from $t_{5}$ to $t_{6}$), only the transmission actions from $t_{0}$ to $t_{5}$ need to be analyzed. Let $k_{h}$ be the change rate of the channel state during $t_{0}$ and $t_{5}$,

(30) $$k_{h}=\left(h_{m}-h_{0}\right)/\left(t_{5}-t_{0}\right),$$

where $h_{m}=h_{t_{5}}$ is the best channel state, and $h_{0}=h_{t_{0}}$ is the worst channel state. At time *t*, the transmission rate is

(31) $$R_{t}=\rho log_{2}\left(1+P_{t}c_{t}\right),$$

where $P_{t}$ is the transmission power, and $c_{t}$ is the channel gain at time *t*. And ρ is the ratio of real transmission rate and upper bound of the achievable transmission rate. The logarithmic channel gain, i.e., the channel state at time *t* is

(32) $$h_{t}=10log_{10}c_{t}.$$

And this equation is equivalent to

(33) $$log_{2}c_{t}=D_{2}h_{t},D_{2}=\frac{log_{2}10}{10}.$$

#### 6.1.2. Energy Cost Minimization Problem

The objective is to minimize energy consumption and deliver a certain amount of data,

(34) $$min\int_{t_{0}}^{t_{5}}P_{t}dt$$

subject to

(35) $$\int_{t_{0}}^{t_{5}}R_{t}dt=\int_{t_{0}}^{t_{5}}\rho log_{2}\left(1+P_{t}c_{t}\right)dt=N_{t_{0}}^{t_{5}}=\lambda \left(t_{5}-t_{0}\right),$$

where $R_{t}$ is the transmission rate at time *t*, $N_{t_{0}}^{t_{5}}$ is the amount of arrival data from $t_{0}$ to $t_{5}$, and λ is the data arrival rate. If the buffer threshold $B_{c}$ is long enough, according to the water-filling algorithm, the optimal transmission power is

(36) $$P_{t}={\left(\;\mu -\frac{1}{c_{t}}\right)}^{+}.$$

#### 6.1.3. Reasonable Buffer Threshold

**Figure 9:**
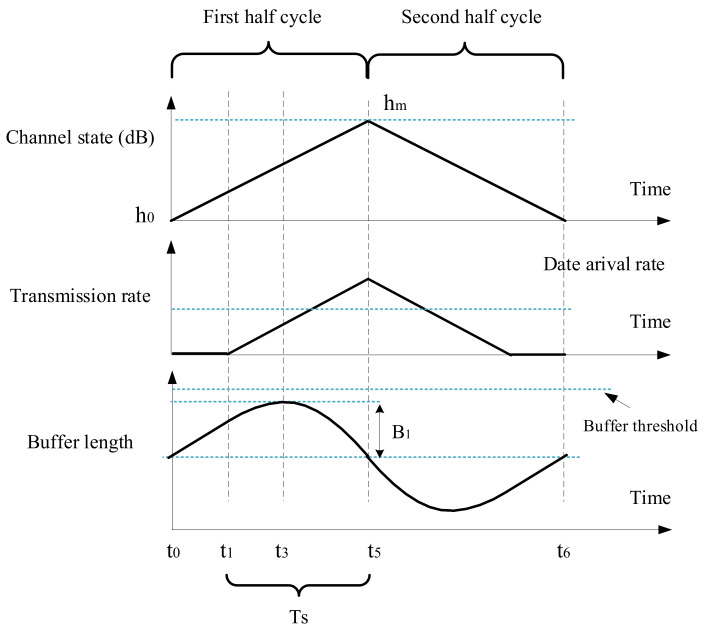
Transmission action when buffer threshold is long enough ($B_{c}\geq 2B_{1}$), for case 1.

The transmission action with sufficiently large buffer threshold is shown in Figure 9. In order to use the optimal transmission power of Equation (36), which is the optimal solution of (34), the buffer threshold should be large enough. In this paper, the minimum buffer threshold required for obtaining the best solution of (34) is called the reasonable buffer threshold $B_{r}=2B_{1}$. And $B_{1}$ will be derived below.

Constraint (35) can be derived as

(37) $$\begin{array}{cc} \int_{t_{1}}^{t_{5}}R_{t}\;dt & =\int_{t_{1}}^{t_{5}}\rho log_{2}\left(1+P_{t}c_{t}\right)dt=\rho \int_{t_{1}}^{t_{5}}log_{2}\left(\mu c_{t}\right)dt\\ \; & =\rho T_{s}log_{2}\left(\mu \right)+\rho \int_{t_{1}}^{t_{5}}D_{2}h_{t}dt\\ \; & ={\rho T}_{s}log_{2}\left(\mu \right){+\rho D}_{2}\left(2h_{m}-k_{h}T_{s}\right)T_{s}/2\\ \; & =N_{t_{0}}^{t_{5}} \end{array},$$

where $T_{s}=t_{5}-t_{1}$ is the length of transmission time during the time of $t_{0}$ to $t_{5}$.

Assume that the transmission rate becomes positive from $t_{1}$, so $P_{t_{1}}=\mu -\frac{1}{c_{t_{1}}}=0$, and

(38) $$log_{2}\mu =-log_{2}c_{t_{1}}={-D}_{2}h_{t_{1}}=k_{h}D_{2}T_{s}-D_{2}h_{m}.$$

Combining (37) and (38), $t_{1}$ can be calculated as

(39) $$t_{1}=t_{5}-T_{s},$$

(40) $$T_{s}^{2}=\frac{2\lambda \left(t_{5}-t_{0}\right)}{k_{h}\rho D_{2}}.$$

And μ is calculated by (38) and (40).

If the buffer threshold $B_{c}$ is long enough, the transmission action is shown in Figure 9. The transmission rate will gradually increase from zero and eventually exceed the data arrival rate. The buffer length will rise to a maximum at $t_{3}$; so, at $t_{3}$, the transmission rate is equal to the arrival rate,

(41) $$R_{t_{3}}=\rho log_{2}\left(\mu c_{t_{3}}\right)=\rho \left(log_{2}\mu +log_{2}c_{t_{3}}\right)=\rho \left(log_{2}\mu +D_{2}\left(h_{0}+k_{h}t_{3}\right)\right)=\lambda .$$

$t_{3}$ can be calculated as

(42) $$t_{3}=\frac{1}{k_{h}}\left(\frac{1}{D_{2}}\left(\lambda /\rho -log_{2}\left(\mu \right)\right)-h_{0}\right).$$

From $t_{0}$ to $t_{3}$, the transmission amount of data is

(43) $$TN\left(t_{1},t_{3}\right)=\int_{t_{1}}^{t_{3}}R_{t}dt\;=\rho \int_{t_{1}}^{t_{3}}log_{2}\left(\mu c_{t}\right)dt=\rho \left(t_{3}-t_{1}\right)log_{2}\left(\mu \right)+\rho D_{2}\left(h_{3}+h_{1}\right)\left(t_{3}-t_{1}\right)/2.$$

From $t_{0}$ to $t_{3}$, the amount of arrival data is $\left(t_{3}-t_{0}\right)\lambda$, so the increment of buffer length is

(44) $$B_{1}=\left(t_{3}-t_{0}\right)\lambda -TN\left(t_{1},t_{3}\right).$$

In order to use the optimal transmit power of Equation (36) from $t_{0}$ to $t_{5}$, the minimum buffer threshold required, i.e., the reasonable buffer threshold is $B_{r}=2B_{1}$. Then, the transmission performance will be derived in three cases, which are divided according to the buffer threshold.

#### 6.1.4. Performance with Large Buffer Threshold

Case 1: If buffer threshold $B_{c}$ is greater than $2B_{1}$, the transmission action is shown in Figure 9. The energy consumption from $t_{0}$ to $t_{5}$ is

(45) $$\begin{array}{cc} E_{1}=\int_{t_{1}}^{t_{5}}\left(\mu -\frac{1}{c_{t}}\right)dt & =\int_{t_{1}}^{t_{5}}\left(\mu -10^{-\frac{h_{t}}{10}}\right)dt\\ \; & =\left(t_{5}-t_{1}\right)\mu +\frac{10}{k_{h}ln10}\left(\frac{1}{c_{t_{5}}}-\frac{1}{c_{t_{1}}}\right) \end{array}.$$

The average transmission energy consumption per unit of data is

(46) $${AE}_{1}=\frac{E_{1}}{\lambda \left(t_{5}-t_{0}\right)}.$$

The average buffer length is

(47) $$AB_{1}=B_{c}-B_{1}.$$

The average transmission delay is

(48) $$AD_{1}=\left(B_{c}-B_{1}\right)/\lambda .$$

#### 6.1.5. Performance with Small Buffer Threshold

**Figure 10:**
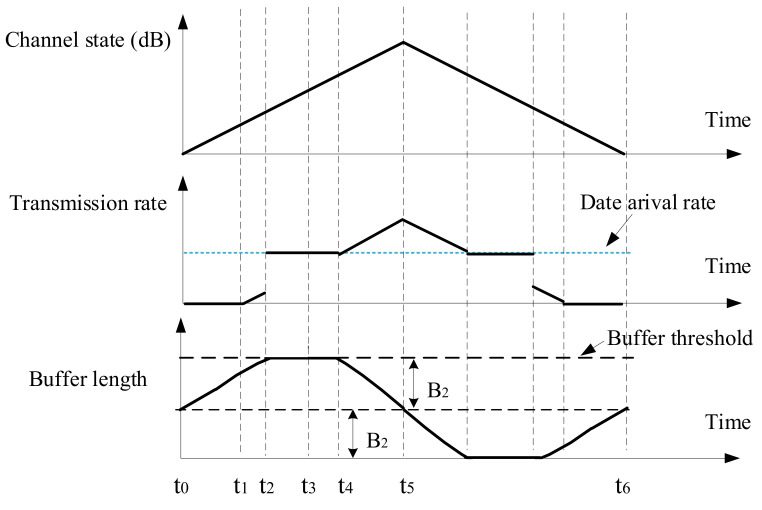
Transmission action when $2\lambda t_{1}\leq B_{c}<2B_{1}$, for case 2.

Case 2: If the buffer threshold $B_{c}$ is less than $2B_{1}$ and greater than $2\lambda t_{1}$, the transmission action is shown in Figure 10. During $t_{0}$ to $t_{1}$, the node does not transmit; during $t_{1}$ to $t_{2}$, the transmission power is ${\left(\;\mu -\frac{1}{c_{t}}\right)}^{+}$; during $t_{2}$ to $t_{4}$, the transmission rate is equal to the data arrival rate; during $t_{4}$ to $t_{5}$, the transmission power is ${\left(\;\mu -\frac{1}{c_{t}}\right)}^{+}$.

From $t_{0}$ to $t_{2}$, the buffer increases from $B_{2}$ to $2B_{2}$,

(49) $$\left(t_{2}-t_{0}\right)\lambda -\int_{t_{1}}^{t_{2}}\rho log_{2}\left(1+P_{t}c_{t}\right)dt=B_{2},$$

where ${B_{2}=B}_{c}/2$. Based on (49), $t_{2}$ can be calculated as

(50) $$t_{2}=\frac{\lambda -\rho log_{2}\left(\mu c_{t_{1}}\right)-\sqrt{{\left(\lambda -\rho log_{2}\left(\mu c_{t_{1}}\right)\right)}^{2}-2{\rho k_{h}D}_{2}\left(B_{2}-t_{1}\lambda \right)}}{\rho k_{h}D_{2}}+t_{1}.$$

Similarly, $t_{4}$ can be calculated as

(51) $$t_{4}=t_{5}-\frac{\rho log_{2}\left(\mu c_{t_{5}}\right)-\lambda -\sqrt{{\left(\rho log_{2}\left(\mu c_{t_{5}}\right)-\lambda \right)}^{2}-2k_{h}\rho D_{2}B_{2}}}{\rho k_{h}D_{2}}.$$

From $t_{0}$ to $t_{5}$, the energy consumption is

(52) $$E_{2}=\int_{t_{1}}^{t_{2}}\left(\mu -\frac{1}{c_{t}}\right)dt+\int_{t_{2}}^{t_{4}}P_{t}dt+\int_{t_{4}}^{t_{5}}\left(\mu -\frac{1}{c_{t}}\right)dt.$$

The three items are calculated as follows:

(53) $$\int_{t_{1}}^{t_{2}}\left(\mu -\frac{1}{c_{t}}\right)dt=\int_{t_{1}}^{t_{2}}\left(\mu -10^{-\frac{h_{t}}{10}}\right)dt=\left(t_{2}-t_{1}\right)\mu +\frac{10}{k_{h}ln10}\left(\frac{1}{c_{t_{2}}}-\frac{1}{c_{t_{1}}}\right).$$

(54) $$\int_{t_{4}}^{t_{5}}\left(\mu -\frac{1}{c_{t}}\right)dt=\int_{t_{4}}^{t_{5}}\left(\mu -10^{-\frac{h_{t}}{10}}\right)dt=\left(t_{5}-t_{4}\right)\mu +\frac{10}{k_{h}ln10}\left(\frac{1}{c_{t_{5}}}-\frac{1}{c_{t_{4}}}\right).$$

From $t_{2}$ to $t_{4}$, the transmission rate is equal to the data arrival rate, so the energy cost is

(55) $$\int_{t_{2}}^{t_{4}}P_{t}dt=\int_{t_{2}}^{t_{4}}\frac{2^{\frac{\lambda }{\rho }}-1}{c_{t}}dt={(2}^{\frac{\lambda }{\rho }}-1)\left(\frac{-10}{k_{h}ln10}\left(\frac{1}{c_{t_{4}}}-\frac{1}{c_{t_{2}}}\right)\right).$$

The average energy consumption per unit of data is

(56) $${AE}_{2}=E_{2}/\left(\lambda \left(t_{5}-t_{0}\right)\right).$$

The average buffer length is

(57) $$AB_{2}=B_{c}/2.$$

The average transmission delay is

(58) $$AD_{2}=B_{c}/2\lambda .$$

**Figure 11:**
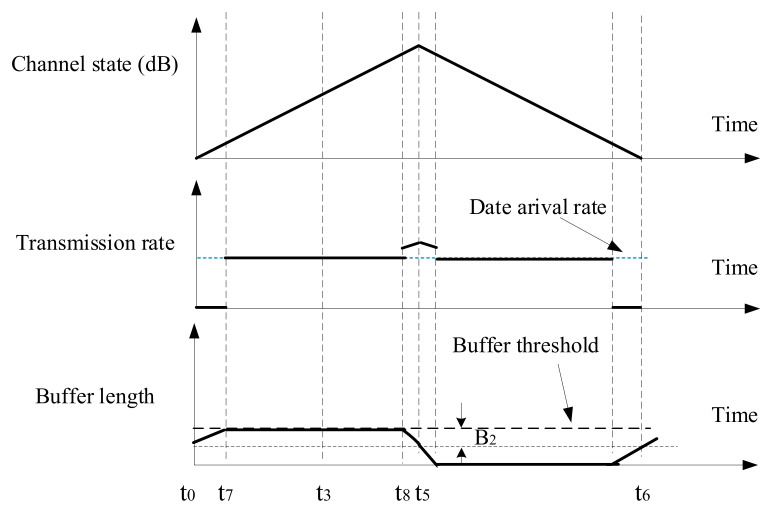
Transmission action when $B_{c}<2\lambda t_{1}$, for case 3.

Case 3: If buffer threshold $B_{c}$ is less than $2\lambda t_{1}$, the transmission action is shown in Figure 11. During $t_{0}$ to $t_{7}$, the node does not transmit; during $t_{7}$ to $t_{8}$, the transmission rate is equal to the data arrival rate; during $t_{8}$ to $t_{5}$, the transmission power is ${\left(\;\mu -\frac{1}{c_{t}}\right)}^{+}$. From $t_{0}$ to $t_{7}$, the amount of arrival data is $B_{2}$, which is half of the buffer threshold $B_{c}$. $t_{7}$ is calculated as

(59) $$t_{7}=B_{2}/\lambda ,$$

where ${B_{2}=B}_{c}/2$. From $t_{8}$ to $t_{5}$, the buffer length decreases from $2B_{2}$ to $B_{2}$. Similar to (51), $t_{8}$ can be calculated as

(60) $$t_{8}=t_{5}-\frac{\rho log_{2}\left(\mu c_{t_{5}}\right)-\lambda -\sqrt{{\left(\rho log_{2}\left(\mu c_{t_{5}}\right)-\lambda \right)}^{2}-2k_{h}\rho D_{2}B_{2}}}{\rho k_{h}D_{2}}.$$

Similar to case 2, the energy consumption is

(61) $$E_{3}=\int_{t_{7}}^{t_{8}}P_{t}dt+\int_{t_{8}}^{t_{5}}\left(\mu -\frac{1}{c_{t}}\right)dt.$$

The two items are calculated as follows:

(62) $$\int_{t_{7}}^{t_{8}}P_{t}dt=\int_{t_{7}}^{t_{8}}\frac{2^{\frac{\lambda }{\rho }}-1}{c_{t}}dt={(2}^{\frac{\lambda }{\rho }}-1)\left(\frac{-10}{k_{h}ln10}\left(\frac{1}{c_{t_{8}}}-\frac{1}{c_{t_{7}}}\right)\right),$$

(63) $$\int_{t_{8}}^{t_{5}}\left(\mu -\frac{1}{c_{t}}\right)dt=\int_{t_{8}}^{t_{5}}\left(\mu -10^{-\frac{h_{t}}{10}}\right)dt=\left(t_{5}-t_{8}\right)\mu +\frac{10}{k_{h}ln10}\left(\frac{1}{c_{t_{5}}}-\frac{1}{c_{t_{8}}}\right).$$

The average energy consumption per unit of data is

(64) $${AE}_{3}=E_{3}/\left(\lambda \left(t_{5}-t_{0}\right)\right).$$

The average buffer length is

(65) $$AB_{3}=B_{c}/2.$$

The average transmission delay is

(66) $$AD_{3}=B_{c}/2\lambda .$$

These quantitative analysis will be calculated in simulation Section 7.4, and impacts of buffer threshold and data arrival rate on communication performance will be presented.

### 6.2. Computational Complexity

The prediction process is composed of large-scale channel state prediction and small-scale channel state prediction. In large-scale channel state prediction, $N_{sch}$ large-scale channel state should be predicted, and, for each prediction, *k*-nearest neighbor prediction algorithm should act on $N_{rl}$ reserved large-scale channel states. So, the computational complexity of the large-scale channel state prediction is $O(N_{sch}N_{rl})$. In small-scale channel state prediction, the number of slots in a large-scale epoch is $N_{large}$, and $N_{sch}N_{large}$ small-scale channel state should be predicted; so, the computational complexity of the small-scale channel state prediction is $O(N_{sch}N_{large})$. In general, $N_{large}\ll N_{rl}$; so, the total complexity of the prediction process is $CC_{1}=O\left(N_{sch}N_{rl}\right)$. This also explains the content in Section 4.1.2, that the amount of storage can be reduced, and the prediction can be speed up, with the decrease of the length of stored historical channel state series.

In terms of the improved genetic algorithm for modulation coding method selection, $N_{c}$ is the number of evolution cycles, and, in each evolution cycle, the fitness of *G* chromosomes should be calculated. Because each chromosome represents modulation and coding modes of $N_{sch}$ large-scale epochs, the performance of $N_{sch}$ large-scale epochs should be summed. So, the computational complexity of the improved genetic algorithm for modulation coding method selection is $CC_{2}=O\left(N_{c}GN_{sch}\right)$. As described in Section 3.3, in a large-scale epoch, the average channel change should be less than a certain threshold, so that the same modulation and coding mode can be allocated for the slots in this large-scale epoch. In this way, the modulation and coding mode only needs to be scheduled once for each large-scale epoch; so, the number of scheduled parameters will decrease, and complexity of scheduling is reduced.Hence, the total complexity of prediction and scheduling schemes is $O(CC_{1}+CC_{2})$.

## 7. Performance Evaluation

### 7.1. Simulation Setting

Simulations are conducted to verify the effectiveness of the double-scale adaptive transmission mechanism in terms of channel prediction and communication performance. The proposed scheme and contrast schemes are evaluated through simulation under the same parameters setting.

The channel state sequence is generated with large-scale and small-scale dynamics. In each slot, the sender transmits one block for 2 s, and the block has 1000 symbols. Unless otherwise specified, the simulation parameters are shown in Table 1.

Multiple sets of channel state series with large-scale and small-scale fluctuations are generated by the superposition of multiple signal sources with the MATLAB toolbox. The generated channel state series combines certain regularity and randomness, which conforms to the channel state fluctuation characteristics described in the literature. For example, if the component of large-scale fluctuation is $f_{t}$, and that of small-scale fluctuation is $g_{t}$, the generated channel state series for simulation can be calculated as

(67) $$\begin{array}{c} h_{t}=f_{t}\;+\;g_{t}+\;C \end{array},$$

where *C* is the coefficient to adjust the mean value of the series so that the channel state approximately satisfies the attenuation and distance relationship of the underwater acoustic channel.

### 7.2. Channel Prediction Performance

Multiple sets of channel state series have been generated for simulation, and each series has 3000 channel measurements. And the results of two sets of series will be shown, which are called Data 1 and Data 2. The fluctuation of Data 1 is relatively regular, while the varying of Data 2 is relatively complicated.

**Figure 12:**
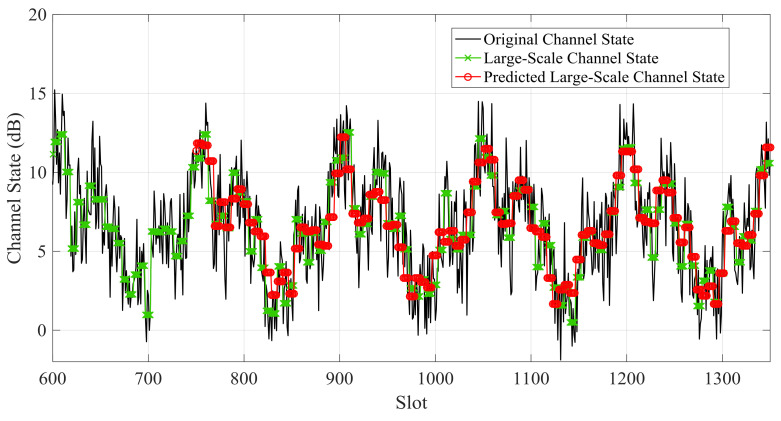
Predicted large-scale channel state and real large-scale channel state of Data 1.

**Figure 13:**
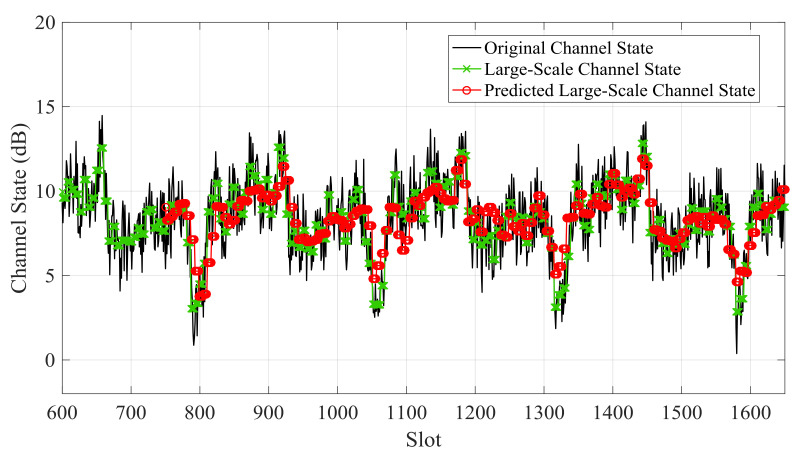
Predicted large-scale channel state and real large-scale channel state of Data 2.

Figure 12and Figure 13 showthe large-scale channel state prediction results of Data 1 and Data 2, respectively. The predicted large-scale channel states are very close to the real large-scale channel states. The root mean square error (RMSE) of large-scale channel state prediction are 1.25 dB for Data 1 and 1.32 dB for Data 2, respectively. The results reveal that the predicted large-scale channel states can reflect the channel trend in the future.

**Figure 14:**
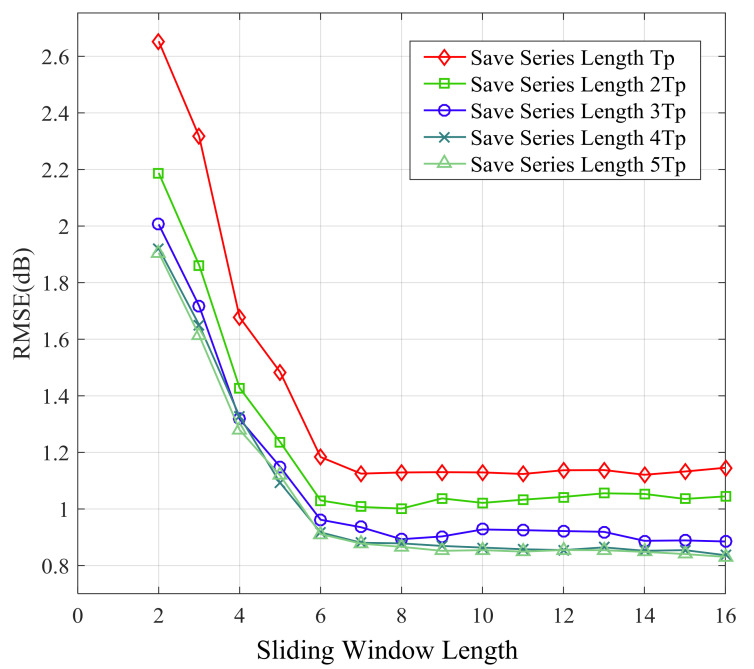
Root mean square error (RMSE) of 1-step ahead prediction with different length of sliding window and stored series.

**Figure 15:**
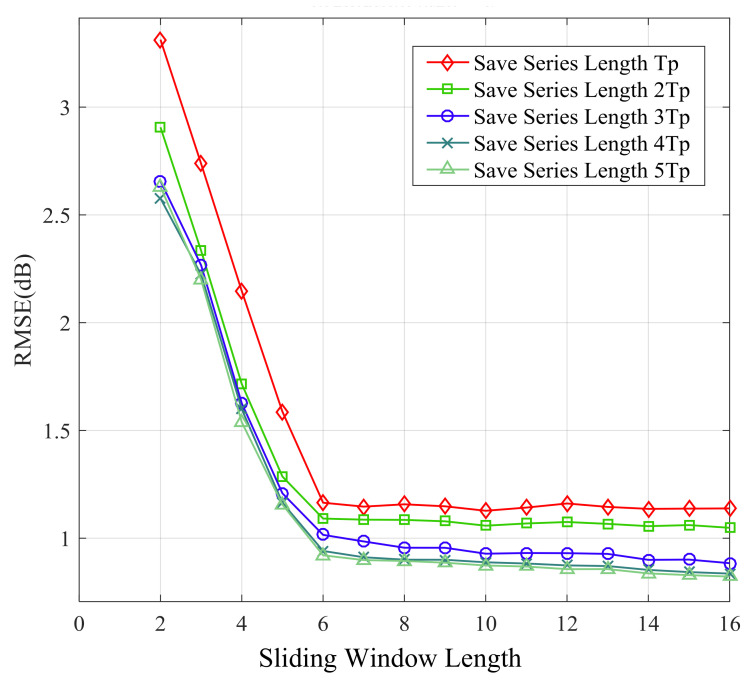
RMSE of 5-step ahead prediction with different length of sliding window and stored series.

**Figure 16:**
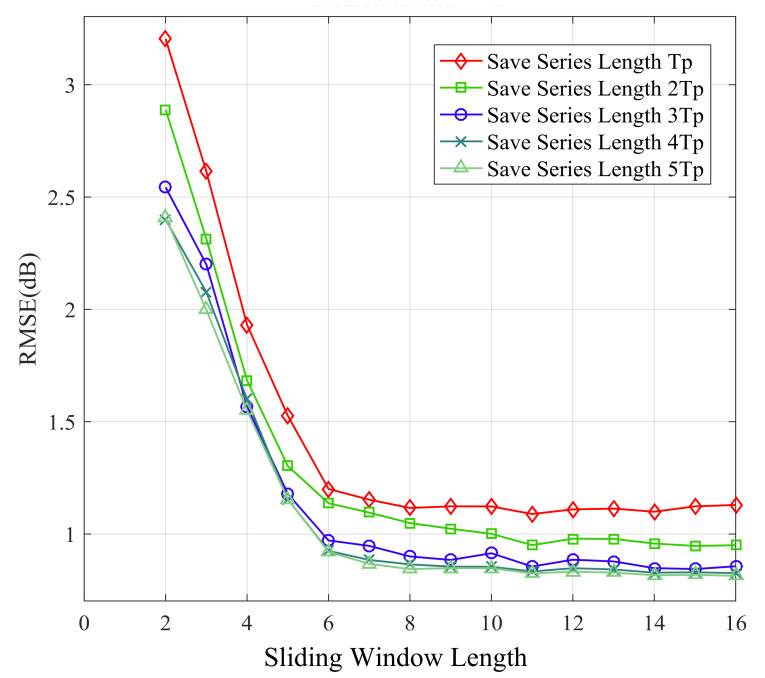
RMSE of 15-step ahead prediction with different length of sliding window and stored series.

**Figure 17:**
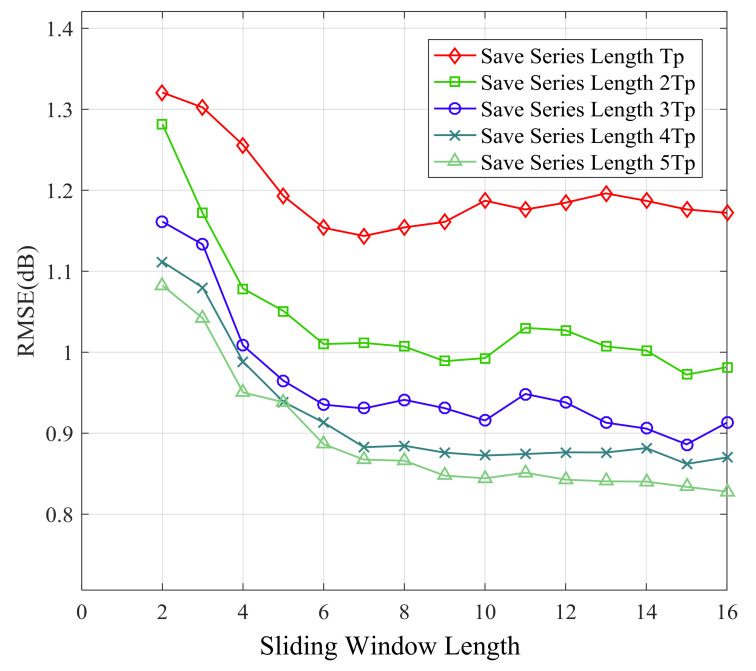
RMSE of 25-step ahead prediction with different length of sliding window and stored series.

Figure 14, Figure 15, Figure 16 and Figure 17 show the large-scale channel prediction performance of Data 1 corresponding to the different lengths of storage series as training set and different lengths of the sliding window. The longer the stored historical series is, the smaller the prediction error is. When the stored historical series exceeds $2T_{p}$, increasing the length of the reserved series has a smaller effect on improving the prediction accuracy. However, the longer the reserved historical series is, the more time the prediction consumes.

As the length of the sliding window increases, the RMSE of prediction decreases gradually. When the length of the sliding window exceeds 6, the RMSE of prediction almost no longer decreases. The effect of the sliding window length on prediction performance can be explained as follows. Each channel state value is affected by previous channel states. In other words, the channel state might display certain time-delay effects on subsequent channel states. Obviously, the larger the vector length is, the more information it contains. Therefore, in order to cover the time-delay effects, the sliding window length *n* (number of selected values) should be large enough. However, overly large length of the sliding window may significantly increase computational complexity for prediction and lead to overfitting, which may cause poor performance [48,49].

**Figure 18:**
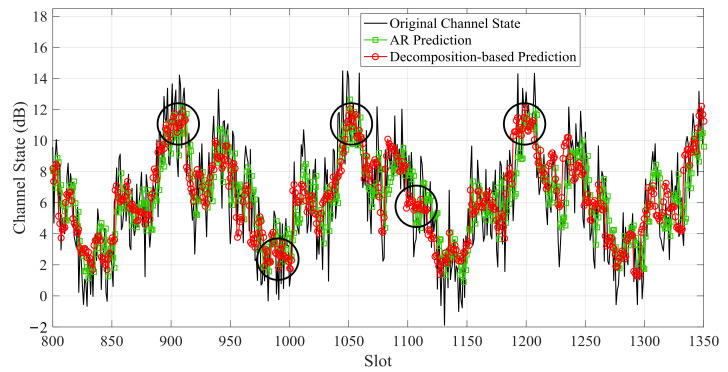
Predicted small-scale channel state of Data 1 by the decomposition-based prediction model and auto-regressive (AR) prediction.

**Figure 19:**
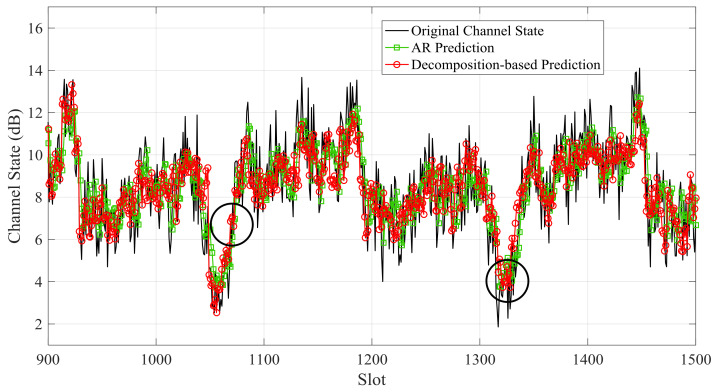
Predicted small-scale channel state of Data 2 by the decomposition-based prediction model and AR prediction.

The small-scale channel state is predicted by the decomposition-based prediction model and AR prediction separately. Figure 18 and Figure 19 show the small-scale channel state prediction results of Data 1 and Data 2, respectively. The RMSEs of decomposition-based prediction is 15.7% and 9.2% lower than that of AR prediction for Data 1 and Data 2, respectively. In the same epoch, channel states of the decomposition-based prediction are relatively stable, while the predicted channel states of the auto-regression prediction fluctuate violently.

**Figure 20:**
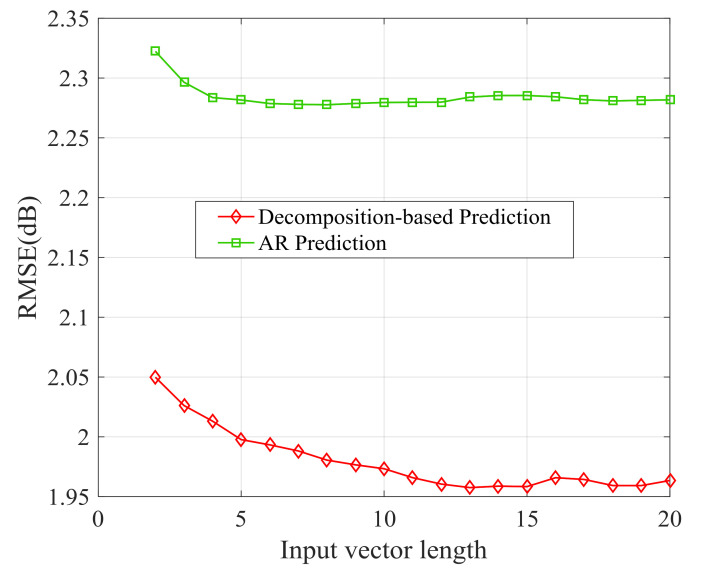
RMSE of small-scale channel state prediction with different input vector length.

In Figure 20, the performances of small-scale channel prediction by two methods are shown for Data 1. The predicted small-scale channel state is used to determine the actual transmission power. RMSE of decomposition-based prediction is smaller than RMSE of AR prediction. The decomposition-based prediction shows better performance.

### 7.3. Data Transmission Performance Comparison

Four modulation and coding modes are used, which is indexed in order of increasing rate, as shown in Table 2. Mode 0 refers to no transmission. The maximal amount of bits that can be carried during one slot is computed based on the transmission mode with the highest data rate, namely Mode 4, as $1000\times 1/2\times log_{2}16$, where 1000 is the number of symbols per block.

The double-scale adaptive transmission mechanism is compared with the contrast schemes in terms of communication performance. Contrast schemes include combination adaptive transmission (AT), channel-based adaptive transmission, and buffer-based adaptive transmission, as shown in Table 3. Each adaptive transmission scheme consists of an adaptive modulation coding strategy and a channel prediction method for power regulation. Channel-based AMC schedules modulation and coding mode according to the predicted channel state of next slot, as shown in Table 4. Buffer-based AMC schedules modulation and coding mode based on the buffer state at the beginning of the transmitting slot, as shown in Table 5. Both the predicted channel state and the buffer state are utilized to determine modulation and coding mode in combination AMC, as shown in Table 6. In Table 4 and Table 6, $\Delta h=h_{max}-h_{min}$.

**Figure 21:**
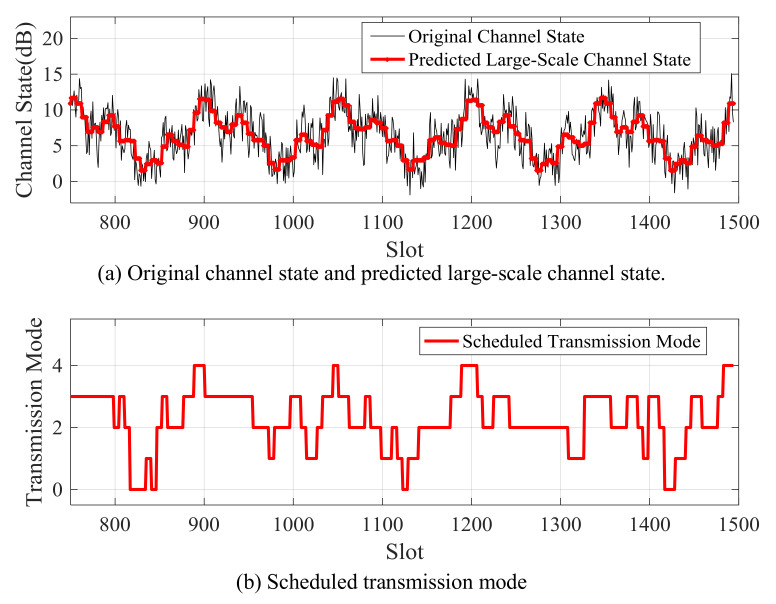
Scheduled transmission mode according to predicted large-scale channel state for Data 1.

**Figure 22:**
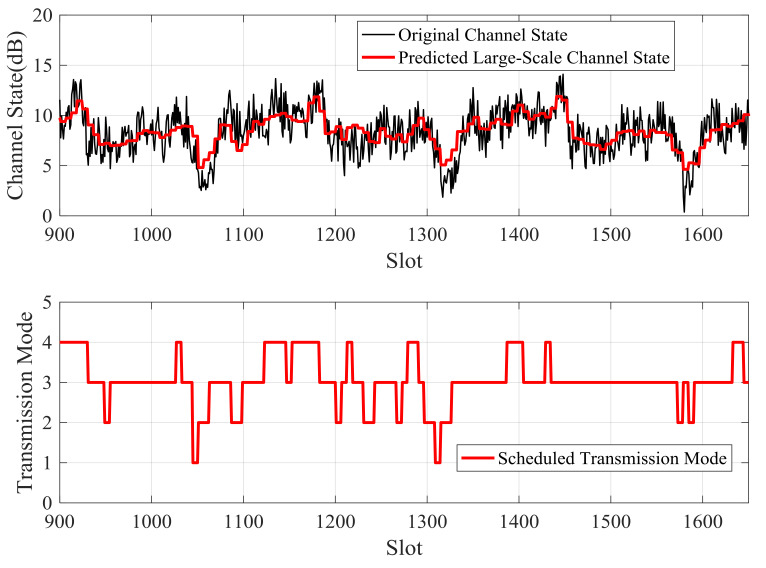
Scheduled transmission mode according to predicted large-scale channel state for Data 2.

When the packet arrival rate is 2 kb/slot, the predicted large-scale channel states and the scheduled future modulation and coding modes of Data 1 and Data 2 are shown in Figure 21 and Figure 22, respectively. When the predicted channel state is good, the higher modulation and coding mode is scheduled; otherwise, the lower modulation and coding mode is adopted. The energy overhead of transmitting a certain amount of data in a good channel state is smaller than that of sending when the channel state is bad. Therefore, in order to improve energy efficiency, the transmitter should send more when the channel is good, and send less or stop sending when the channel is bad.

**Figure 23:**
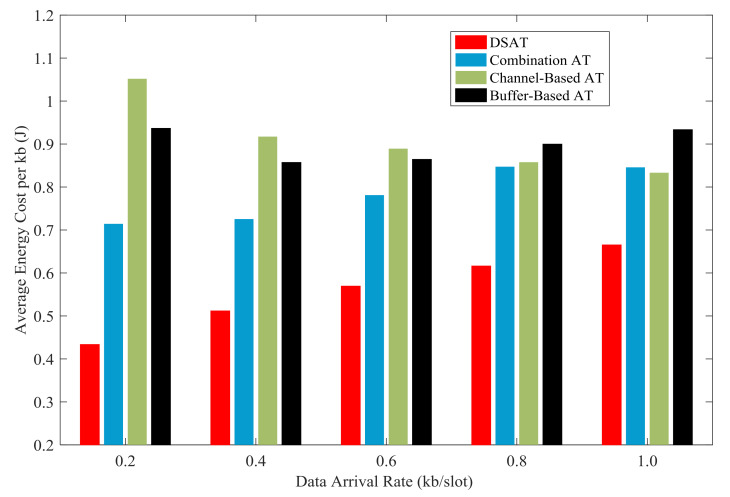
Average energy cost per kb of for comparative strategies (Data 1).

**Figure 24:**
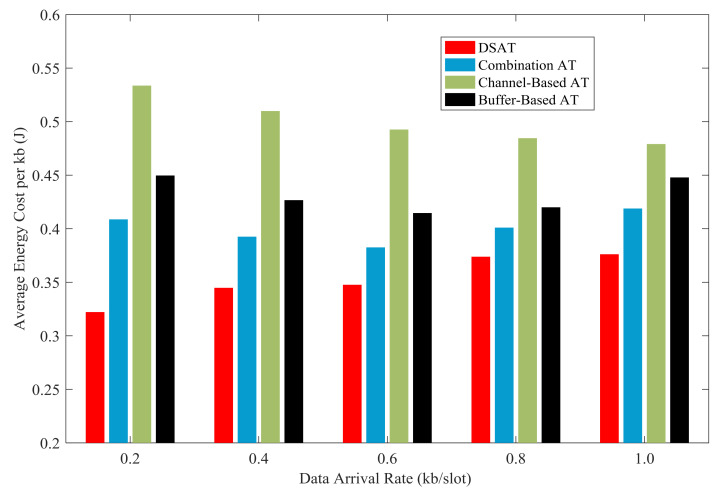
Average energy cost per kb for comparative strategies (Data 2).

In Figure 23 and Figure 24, the energy cost per kb of all schemes are compared with data arrival rate from 0.2 kb/slot to 1.0 kb/slot. Overall, compared with the comparison algorithm, the energy cost of double-scale adaptive transmission is the lowest. In other words, double-scale adaptive transmission achieves higher energy efficiency than contrast schemes. The good energy efficiency results from the reasonable schedule of modulation and coding mode, as shown in Figure 21.

**Figure 25:**
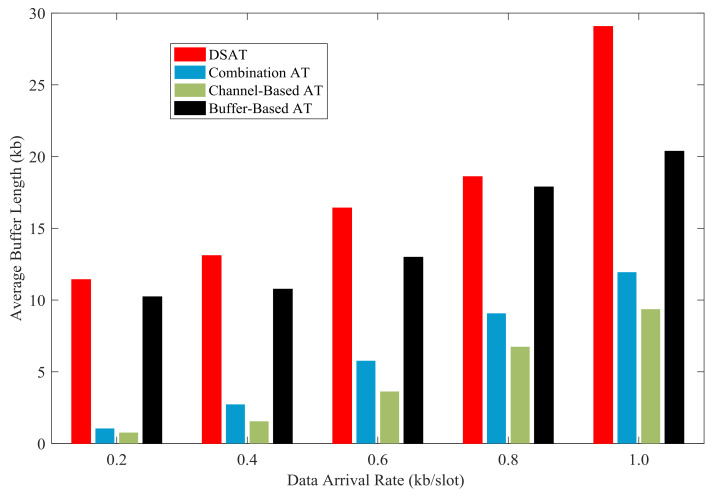
Average buffer length for comparative strategies (Data 1).

**Figure 26:**
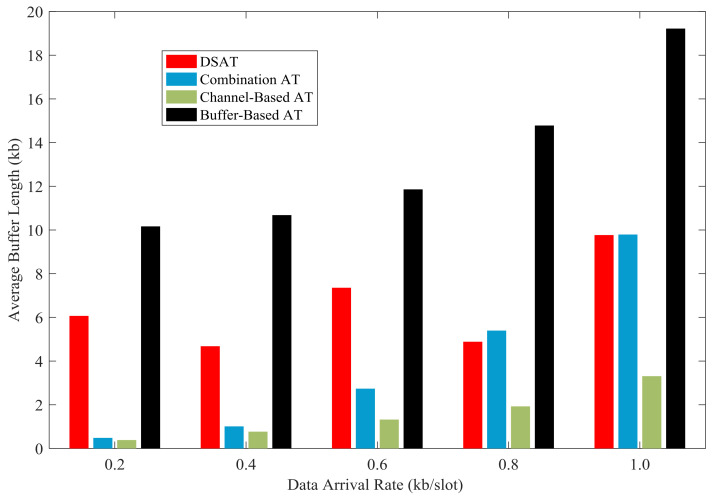
Average buffer length for comparative strategies (Data 2).

In Figure 25 and Figure 26, the average buffer length of each scheme is shown with data arrival rate from 0.2 kb/slot to 1.0 kb/slot. The average buffer length of DSAT is longer than that of other schemes. This reflects that the transmitter often waits until the channel is good. With the increase of the data arrival rate, the average buffer length of each scheme increases.

**Figure 27:**
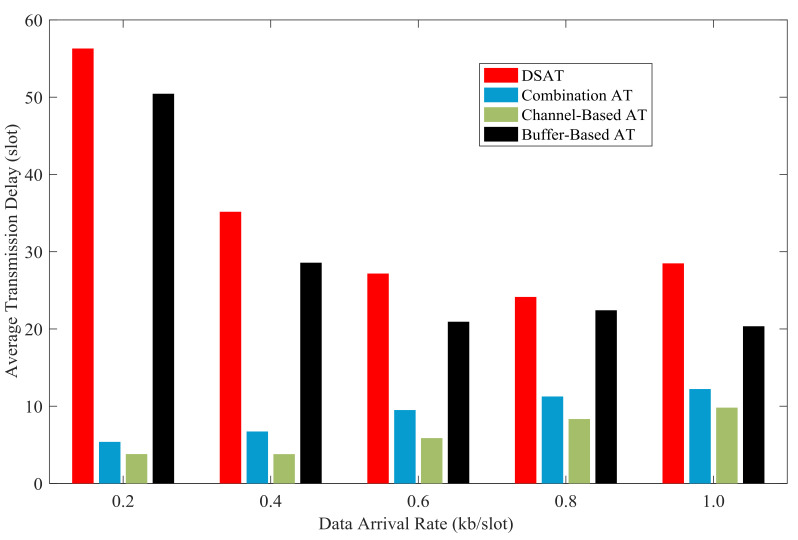
Average transmission delay for comparative strategies (Data 1).

**Figure 28:**
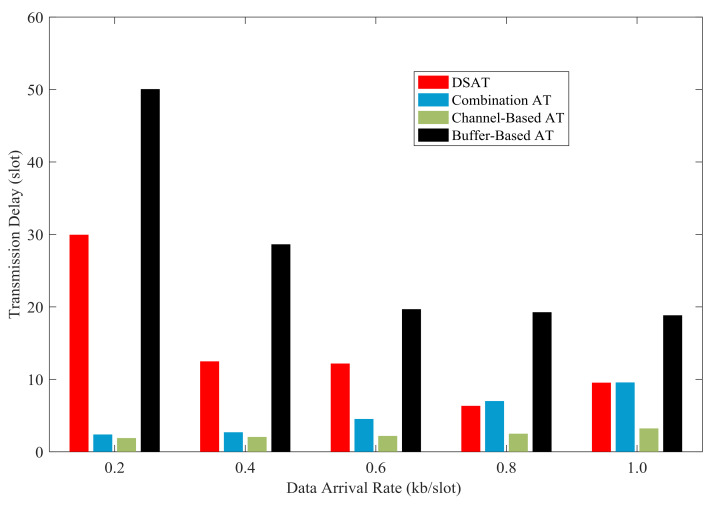
Average transmission delay for comparative strategies (Data 2).

In Figure 27 and Figure 28, the average transmission delay of each scheme is shown with data arrival rate from 0.2 kb/slot to 1.0 kb/slot. The average transmission delay of DSAT is longer than that of other schemes. The average transmission delay of DSAT decreases with the increase of data arrival rate, and the reason is as follows. When the data arrival rate is very low, packets stay in the buffer until the channel state gets better, which makes the average transmission delay relatively long. With the increase of data arrival rate, some packets have to be transmitted, although the channel state is not good, in order to send a predetermined amount of data. Thus, the average transmission delay will be reduced.

### 7.4. Influence of Buffer Threshold

Through the simulation of Data 1, the impacts of buffer threshold on communication performance is obtained. Based on the quantitative theoretical analysis of transmission performance which is described in Section 6.1, the theoretical results of the impact of buffer threshold on communication performance are obtained. The parameters of the linearly varying channel state series used for theoretical analysis is as follows: $h_{0}=1$ dB, $h_{m}=15$ dB, $t_{5}-t_{0}=30\;slots$. Simulation results and theoretical analysis are presented.

**Figure 29:**
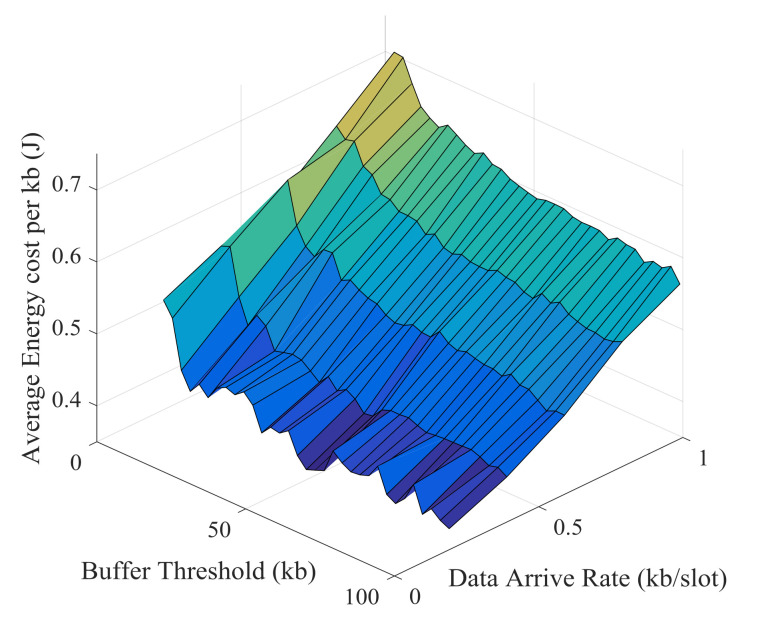
Simulation results of the impact of buffer threshold and data arrival rate on the average energy cost.

**Figure 30:**
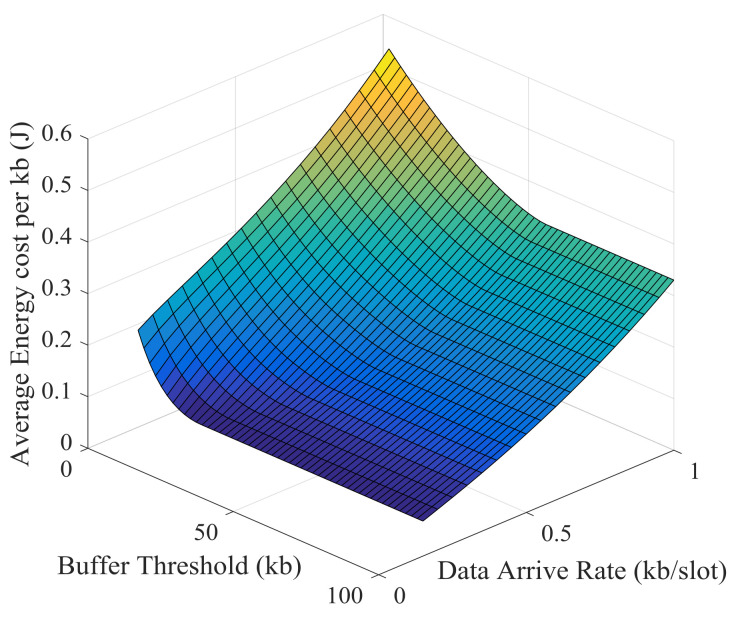
Theoretical results of the impact of buffer threshold and data arrival rate on the average energy cost.

Simulation results and theoretical analysis of the impact of buffer threshold and data arrival rate on the average energy cost per kb are shown in Figure 29 and Figure 30. The average energy cost decreases as the buffer threshold increases, but the decrease becomes slower and slower. After the buffer threshold exceeds a critical value, the average energy cost hardly decreases. This critical value is positively correlated with the data arrival rate. The buffer threshold can be set based on this critical value to improve energy efficiency and prevent excessive delay.

**Figure 31:**
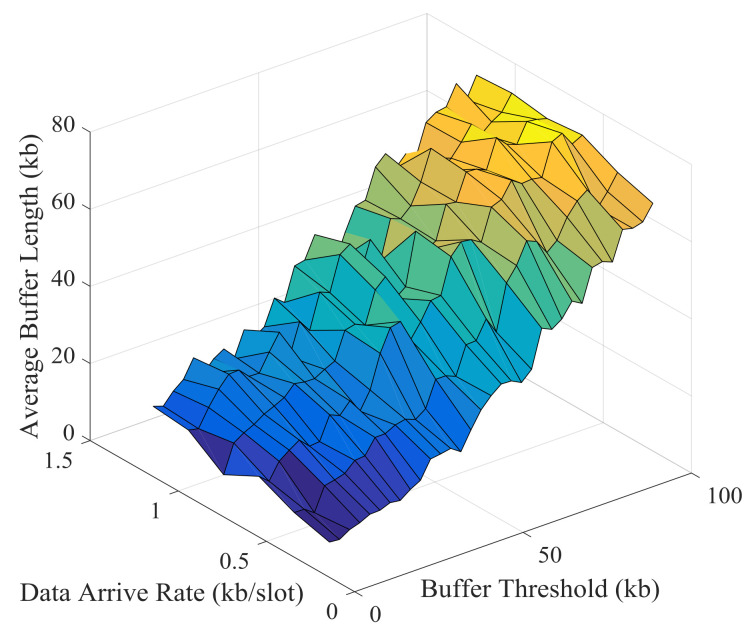
Simulation results of the impact of buffer threshold and data arrival rate on the average buffer length.

**Figure 32:**
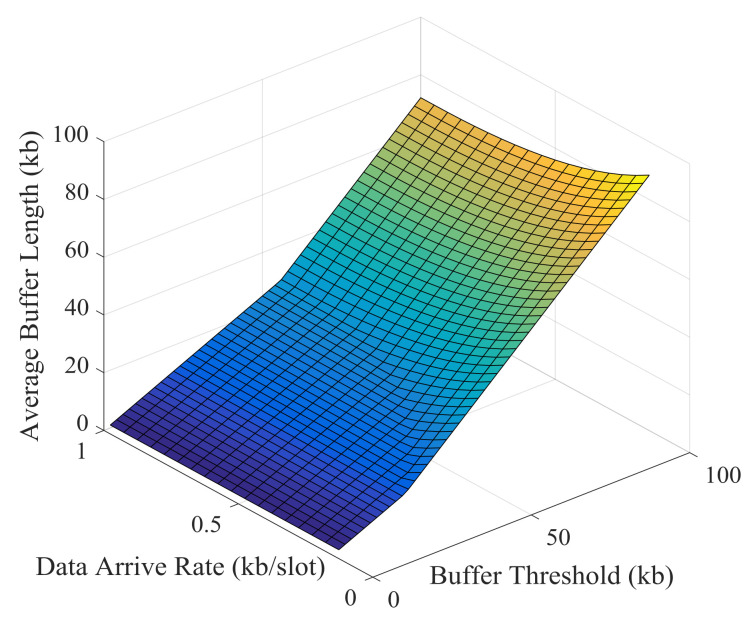
Theoretical results of the impact of buffer threshold and data arrival rate on the average buffer length.

Simulation results and theoretical analysis of the impact of buffer threshold and data arrival rate on the average buffer length are shown in Figure 31 and Figure 32. The average buffer length increases rapidly with the increase of buffer threshold. Obviously, there is a strong correlation between buffer threshold and average buffer length, which can be obtained from theoretical analysis.

**Figure 33:**
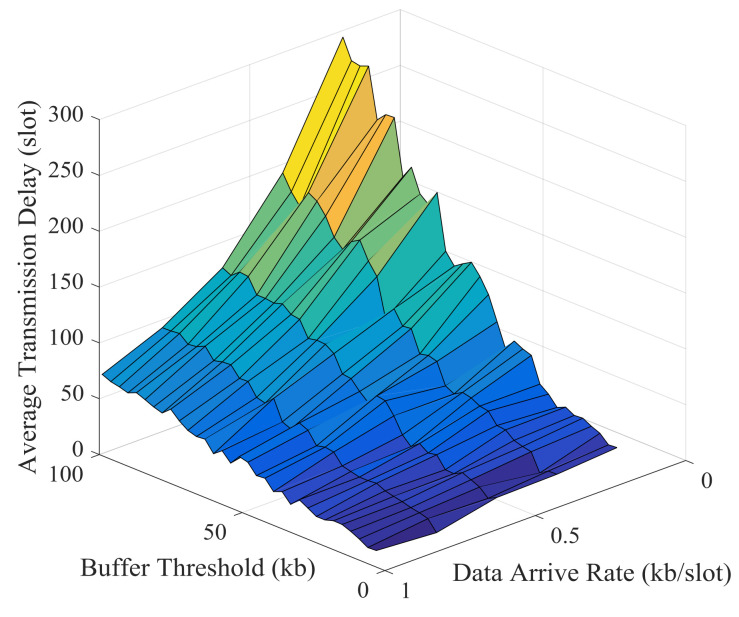
Simulation results of the impact of buffer threshold and data arrival rate on the average transmission delay.

**Figure 34:**
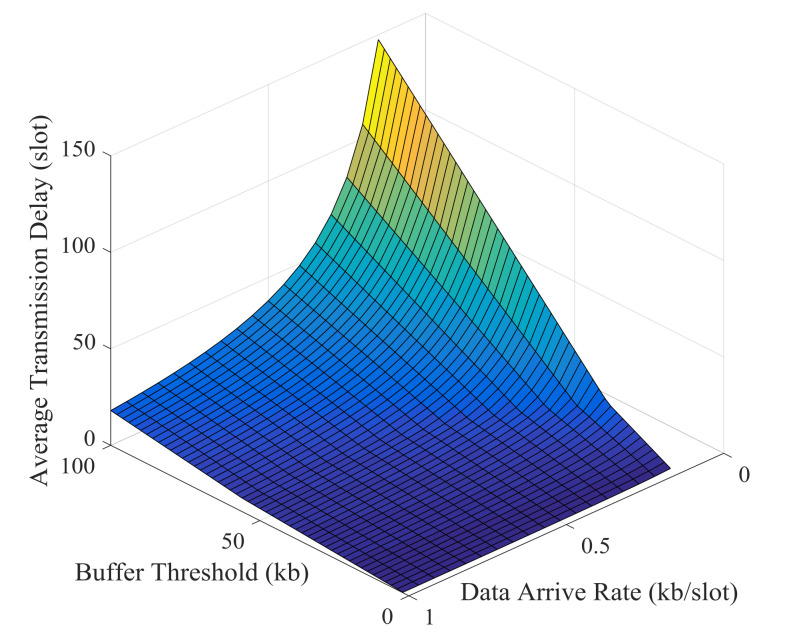
Theoretical results of the impact of buffer threshold and data arrival rate on the average transmission delay.

Simulation results and theoretical analysis of the impact of buffer threshold and data arrival rate on the average transmission delay are shown in Figure 33 and Figure 34. When the data arrival rate is very low, the average delay increases rapidly with the increase of the buffer threshold; when the data arrival rate is high, the average delay increases relatively slowly as the buffer threshold increases. With a certain buffer threshold, the transmission delay decreases as the data arrival rate increases. And this is consistent with the result of theoretical analysis.

## 8. Conclusions

In this paper, a double-scale adaptive transmission mechanism has been proposed for UASNs with time-varying channels. Firstly, the historical channel state series has been decomposed into large-scale and small-scale series, which can then be predicted by a novel *k*-nearest neighbor search algorithm with sliding window and auto-regressive algorithm, respectively. Since only a few historical channel state series are needed for channel prediction, the proposed mechanism can ensure the prediction performance with a greater reduction of the computation complexity and the storage size. Then, an energy-efficient transmission algorithm is designed to solve the problem of long-term modulation and coding optimization, and an improved genetic algorithm is designed to accelerate the convergence speed. With the theoretical analysis for the transmission impact of buffer threshold adopted in our proposed mechanism, we have optimized the selection of buffer length. Numerical simulation results show that the proposed methods achieve good performance in terms of channel prediction and energy efficiency. The predicted large-scale channel states can reflect the channel trend in the future, and the adaptive transmission mechanism can significantly reduce the energy consumption of communication. Meanwhile, the simulation results of buffer threshold impact on average energy cost and average transmission delay are consistent with the theoretical analysis. For our future work, we will investigate the combination of the double-scale adaptive transmission mechanism and media access control protocol to further improve the overall performance of underwater acoustic sensor networks.

## Figures and Tables

**Table 1:** Simulation parameters.

Parameter	Value
Transmission distance	1 km
Acoustic speed	1500 m/s
Carrier frequency	10 kHz
bandwidth	5 kHz
Time slot	2 s
Block size	1000 symbols
Buffer Capacity	100 kb

**Table 2:** Modulation and coding modes.

Transmission Mode	Modulation Methods	Coding Rate
Mode 0	stop transmitting	
Mode 1	BPSK	1/2
Mode 2	QAM	1/2
Mode 3	QAM	3/4
Mode 4	16QAM	1/2

**Table 3:** Comparative schemes.

Adaptive Schemes	Name	AMC Strategy	Channel Prediction Method
scheme 1	DSAT	Energy-Efficient Transmission	Decomposition-based Prediction algorithm
scheme 2	Combination AT	Combination AMC	AR Prediction algorithm
scheme 3	Channel-Based AT	Channel-based AMC	AR Prediction algorithm
scheme 4	Buffer-Based AT	Buffer-based AMC	AR Prediction algorithm

**Table 4:** Channel-based adaptive modulation and coding (AMC).

Channel State	Mode
*h*≤$h_{min}+0.2\Delta h$	stop
$h_{min}+0.2\Delta h$ < *h*≤$h_{min}+0.4\Delta h$	Mode 1
$h_{min}+0.4\Delta h$ < *h*≤$h_{min}+0.6\Delta h$	Mode 2
$h_{min}+0.6\Delta h$ < *h*≤$h_{min}+0.8\Delta h$	Mode 3
*h* > $h_{min}+0.8\Delta h$	Mode 4

**Table 5:** Buffer-based AMC.

Buffer State	Mode
Buffer = 0 kb	stop
0 kb < Buffer ≤ 5 kb	Mode 1
5 kb < Buffer ≤ 15 kb	Mode 2
15 kb < Buffer ≤ 25 kb	Mode 3
Buffer > 25 kb	Mode 4

**Table 6:** Combination AMC.

Condition	Buffer ≤ 10 kb	10 kb < Buffer ≤ 30 kb	Buffer > 30 kb
*h* > $h_{min}+2\Delta h/3$	Mode 2	Mode 3	Mode 4
$h_{min}+\Delta h/3$ < *h* ≤ $h_{min}+2\Delta h/3$	Mode 1	Mode 2	Mode 3
*h*≤$h_{min}+\Delta h/3$	stop	Mode 1	Mode 2

## Data Availability

Data sharing not applicable.
